# Bioinformatics strategies and biomarker refinement using high-throughput transcriptome data in transplantation

**DOI:** 10.3389/fbinf.2026.1677453

**Published:** 2026-04-08

**Authors:** Oliver P. Günther, Karen R. Sherwood, Franz Fenninger, Robert F. Balshaw, Andreas Scherer, Zsuzsanna Hollander, Raymond Ng, Janet Wilson-McManus, W. Robert McMaster, Bruce M. McManus, Paul A. Keown

**Affiliations:** 1 Günther Analytics, Vancouver, BC, Canada; 2 Department of Pathology and Laboratory Medicine, University of British Columbia, Vancouver, BC, Canada; 3 PROOF Centre of Excellence, Vancouver, BC, Canada; 4 College of Community and Global Health, University of Manitoba, Winnipeg, MB, Canada; 5 Institute for Molecular Medicine Finland, FIMM University of Helsinki, Helsinki, Finland; 6 Department of Computer Science, University of British Columbia, Vancouver, BC, Canada; 7 Infection & Immunity Research Centre, University of British Columbia, Vancouver, BC, Canada; 8 Medical Genetics, University of British Columbia, Vancouver, BC, Canada; 9 James Hogg iCAPTURE Centre, Vancouver, BC, Canada; 10 Department of Medicine, University of British Columbia, Vancouver, BC, Canada

**Keywords:** acute rejection, bioinformatics, biomarkers, classification, gene expression, kidney transplantation, whole blood

## Abstract

**Introduction:**

Renal transplantation is the treatment of choice for kidney failure, but most transplants fail prematurely and barely half of recipients survive with a functioning graft for more than a decade. Strategies to induce operational tolerance are therefore at the cutting edge of transplant research, exploiting the dynamic plasticity of the immune system to recapitulate neonatal ontogeny and permit gradual withdrawal of immune suppression. We have shown that whole blood gene expression is profoundly altered in uremia and following graft implantation, and that changes in the blood transcriptome are characteristic of rejection injury. But deriving simple, robust and parsimonious classifiers presents challenges, and pre-filtering methods of varying stringency have been proposed to enhance predictive accuracy.

**Methods:**

We re-analyzed our previous data documenting transcriptome changes in rejection using a case-control design to compare analytical strategies in subjects with or without biopsy-proven rejection. Five pre-filtering methods and eight multivariate classification methods were evaluated using multiple partition nested cross-validation to obtain unbiased estimation of classifier performance.

**Results:**

The most permissive strategy identified 800 unique genes and the most restrictive identified 71 nested genes differentially expressed in rejectors of which 31%–45% were downregulated and 55%–69% were upregulated, reflecting neutrophil degranulation, regulated necrosis, programmed cell death, pyroptosis, interleukin signaling and other functional pathways. Of the ten most common genes or probe-sets over all panels, nine were increased in BCAR.

**Discussion:**

No individual combination of methods presented superior performance among all those considered although the PAM and XGBoost classifiers were more resistant to over-fitting. It is therefore advisable to apply multiple analytical combinations and compare performances in transcriptome analysis. In limited resource situations, evaluation of at least two complementary classifiers with fixed pre-filter and ranking methods is advisable. For small panel size constraints, feature-selecting methods like PAM or EN could be considered.

## Graphical Abstract

**Figure:**
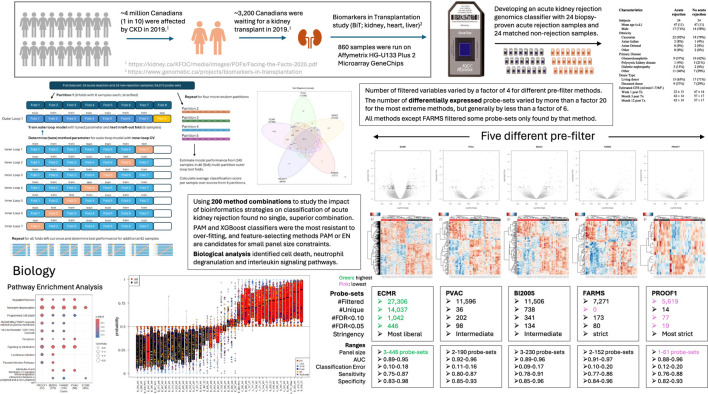
Diagram summarizing transplantation landscape in Canada, patient characteristics and sample size, multi-partition, nested cross-validation framework, pre-filter methods, concordance, differential expression, classification performance and pathway enrichment analysis results.

## Introduction

Chronic kidney disease (CKD) is a debilitating disorder with profound medical and societal consequences that affects almost 10% of the world’s population (Chen et al., 2019; Francis et al., 2024; GBD Chronic Kidney Disease Collaboration, 2020). The pleomorphic clinical and biological manifestations of uremia reflect complex disorders of cellular biology, with alterations in innate and adaptive immunology causing simultaneous inflammation and immune deficiency (Scherer et al., 2013; Meijers et al., 2024; Song et al., 2025). Renal transplantation is the treatment of choice for kidney failure but, though advances in surgery and medicine have improved safety and success, most transplants still fail prematurely and only half of recipients survive with a functioning graft for more than a decade (Hariharan et al., 2021; Levy et al., 2014). Graft rejection remains a leading cause of premature failure (Betjes et al., 2022). The recipient response to the allograft reflects a sophisticated concert of coordinated cognate interactions involving the innate and adaptive immune systems in which either cellular or antibody-mediated injury may predominate (Gónther et al., 2009; Naesens et al., 2024). Continuous or repeated episodes of inflammation lead to chronic mesenchymal change, vascular luminal narrowing and organ fibrosis characteristic of chronic rejection (Cornell et al., 2008).

Potent immunosuppressive therapy is required to suppress the rejection response but is toxic, expensive and challenging to personalize and is part of the barrier to long-term success (Khan et al., 2025; Rostaing et al., 2023). Strategies to induce operational tolerance are therefore at the cutting edge of transplant research, combining donor antigen and cellular therapies in the first weeks post-transplant when the dynamic plasticity of the immune system may recapitulate neonatal ontogeny and permit gradual withdrawal of immune suppression. We have shown that CKD causes profound changes in the human transcriptome (Scherer et al., 2013) with further alterations following graft implantation, during rejection and on treatment which then return towards normal in quiescence (Gónther et al., 2009; Günther et al., 2011). We plan to confirm these initial findings using rapid, long nanopore RNA sequencing which offers the potential for real-time monitoring of these dynamic transcriptomic changes to guide the use of personalized therapies for tolerance induction.

We have shown that whole blood gene expression of recipients is profoundly altered following graft implantation, and that changes in the blood transcriptome occur which are characteristic of rejection injury (Gónther et al., 2009). The biological functions of the differentially expressed genes encompass major biological categories of cellular processes related to immune signal transduction, cytoskeletal reorganization and apoptosis, and emphasize the participation of the cytokine-activated Jak-Stat pathway and interferon-γ signaling in lymphocyte activation proliferation, chemotaxis and adhesion (Gónther et al., 2009). We have observed that these changes in the blood transcriptome are paralleled by characteristic changes in the plasma proteome, and that plasma proteins that encompassed processes related to inflammation, complement activation, blood coagulation, and wound repair exhibited significantly different relative concentrations between patient cohorts with and without biopsy-proven acute rejection (BCAR) (Cohen et al., 2010). Longitudinal monitoring over the first 3 months post-transplant shows that these alterations in the human transcriptome and proteome peak at the time of graft rejection, decline after treatment, then return to baseline throughout the subsequent period of quiescence (Gónther et al., 2009; Günther et al., 2011).

Deriving simple, robust and parsimonious classifiers which can be utilized broadly for clinical purposes presents challenges, however. Different pre-filtering methods of varying stringency have been proposed to enhance the accuracy of the initial microarray data, and a variety of unsupervised and supervised approaches have been proposed for analysis (Wiltgen and Tilz, 2007; Natsoulis et al., 2005; Allison et al., 2006; Mieczkowski et al., 2010; Dai et al., 2005). Pre-filtering methods may range in stringency from permissive models, such as the ones we have previously employed, to restrictive algorithms returning progressively fewer probe-set values (Gónther et al., 2009; Clevert et al., 2011; Lu et al., 2011). Class comparison may be performed using techniques such as linear models for microarray analysis (LIMMA) (Limma et al., 2005; Ritchie et al., 2015) or statistical analysis of microarrays (SAM) (Tusher et al., 2001), with visualization using hierarchical clustering and principal component analysis (PCA). Subsequent inference, filtering and ranking of the gene list may be conducted using false discovery rate (FDR), fold change (FC) or a combination of these parameters (Allison et al., 2006).

Unsupervised methods alone are valuable for class comparison and discovery, but do not provide decision rules for classification (Natsoulis et al., 2005; Allison et al., 2006). A number of supervised methods exist for class prediction, and encompass linear discriminant analysis (LDA, SDA), generalized linear models (GLM), elastic nets (EN), support vector machines (SVMs), shrunken centroids (also termed prediction analysis of microarrays, PAM), decision trees and ensemble classifiers such as random forest (RF) or extreme gradient boosting (XGBoost) (Natsoulis et al., 2005; Friedman et al., 2010; Tibshirani et al., 2002; Breiman, 2001; Chen and Guestrin, 2016). These require a supervised learning process with a training dataset. Clinical or histopathological descriptors are employed to define phenotypic classes, and each observation is classified using a mathematically derived separation function. Supervised methods are highly flexible, and employ linear or non-linear separation functions, that include all or subsets of variables in this process (Natsoulis et al., 2005; Allison et al., 2006). Supervised methods for class-prediction require a training phase during which the classification model is learned based on training data. The trained classifier should generally be tested in an independent dataset for unbiased classification performance estimation.

In the absence of uniform guidelines, a range of strategies have been employed for biomarker development (Chen et al., 2011; Chang et al., 2011). We have therefore drawn on our prior data and applied several of these approaches to our previous studies of gene expression in renal transplantation in order to develop or confirm robust and parsimonious signatures that have a high performance for the diagnosis of acute graft rejection. We show here the variety of signatures that are produced by individual bioinformatic approaches, the relevant biological pathways invoked by each, and discuss practical implications.

## Materials and methods

Study subjects: All subjects receiving a renal transplant at Vancouver General Hospital or St. Paul’s Hospital, Vancouver, Canada from 1 January 2005 to 31 December 2009 were invited by the principal investigator or designate to participate in the study which was approved by the UBC Clinical Research Ethics Board. Those who agreed and provided signed informed and witnessed consent were enrolled and were followed routinely by the transplant program team throughout the next 3 years of their clinical course (Gónther et al., 2009). Patients received a standardized treatment protocol including basiliximab 20 mg i.v. on days 0 and 4, methylprednisolone 125 mg iv on the day of transplantation tapering to zero by day 3 post-transplant, tacrolimus 0.075 mg/kg b.i.d and mycophenolate 1,000 mg b.i.d. Tacrolimus concentrations were measured by tandem mass spectrometry, and the dose was adjusted to achieve 12-h trough levels of 8–12 ng/mL in month 1, 6–9 ng/mL in month 2, and 4–8 ng/mL thereafter. Graft rejection was diagnosed using standard clinical and laboratory parameters, confirmed by biopsy, and graded according to the Banff working classification of renal allograft pathology (Racusen et al., 1999). Banff categories 2 and 4 (antibody-mediated or acute/active cellular rejection) were considered significant. Subjects with borderline changes (Category 3) were analyzed separately. All demographic, clinical, diagnostic and therapeutic data were recorded longitudinally in an electronic database and there was no patient loss to follow-up during the study.

Study design: Blood samples during the first year were obtained in PAXgene™ tubes immediately prior to transplantation, at 0.5, 1, 2, 3, 4, 8, 12, 26, and 52 weeks post-transplant, and at the time of suspected rejection. Graft tissue was obtained pre-transplant and at the time of all biopsies performed post-transplant. All samples were stored at −80^o^C until required for analysis. Blood samples from normal healthy controls, treated identically, served as comparators (Gónther et al., 2009). The study employed a case-control design to compare differential gene expression in subjects with or without BCAR (Etminan and Samii, 2004). To ensure precise homogeneous phenotypes, patients were considered eligible for analysis if they were less than 75 years of age; did not have pre-transplant immunosuppression or immunological de-sensitization; received an AHG-CDC crossmatch negative kidney transplant from a deceased or non-HLA-identical living donor; did not receive depleting antibody induction therapy; were able to receive oral immunosuppression, and had no evidence of infection, disease recurrence, and other major co-morbid events. Cases with BCAR diagnosed during the first 12 months post-transplant were matched as closely as possible for age, sex, degree of sensitization, organ source and date of transplantation with controls who had no evidence of clinical or BCAR during the period of follow-up, and peripheral blood samples from cases and controls were compared at identical time points.

Gene expression: Total RNA was extracted using a PAXgene™ Blood RNA kit, and integrity and concentration determined using the Agilent 2,100 BioAnalyzer (Agilent Technologies, Palo Alto, CA). Gene expression was analyzed at the CAP/CLIA certified Microarray Core Laboratory, Genome Core at the Children’s Hospital, Los Angeles, CA, using Affymetrix Human Genome U133 Plus 2.0 arrays. Quality of the samples, hybridization, chips and scanning was reviewed using the tools in the *BioConductor* packages *affy* version 1.16.0 and *affyPLM* version 1.14.0. Background correction, normalization and summarization of the data were performed using the Robust Multi-array Average (RMA) method (Wiltgen and Tilz, 2007) with more details in Supplementary Material. Expression values were analyzed on the log-base 2 scale.

Pre-filtering: Five pre-filtering methods were employed, ranging from most permissive to most restrictive: Empirical Central Mass Range (ECMR) (Günther et al., 2012) which retains half of the 54,613 probe-sets on the Affymetrix GeneChip with the largest inter-quartile range, PVAC (Lu et al., 2011) which only retains probe-sets that show consistent probe-level expression, BI2005 (Sing et al., 2005) which sets all probe-set values of less than 5 to 5 and then selects only probe-sets that have a maximum log2-expression of at least 7.25 across all analysis samples, FARMS (Clevert et al., 2011) which selects only informative probe-sets based on Bayesian factor analysis and PROOF1 (Shin et al., 2014), a semi-automated, biologically and clinically motivated approach which combines different criteria, including gene expression cutoffs, Affymetrix annotations and time-course expression data to select robust genes. All pre-filters were applied pre-analysis to compile five different analysis data sets before univariate and multivariate ranking and filtering steps were applied within a cross-validation framework. More details about the ECMR pre-filter are provided in the Supplements.

Class comparison: Differential expression of probe-sets between subjects with or without BCAR was determined using Linear Models for Microarray Data (LIMMA) in Bioconductor (Ritchie et al., 2015), including empirical Bayes shrinkage of the standard error towards a common value and multiple hypothesis testing adjustments based on Benjamini-Hochberg’s method. Data visualization was performed using volcano diagrams and hierarchical clustering.

Ranking and filtering: Results returned by the univariate LIMMA analysis were used to rank and filter probe-sets by adjusted p-value (false discovery rate [FDR]) and fold-change, followed by optional multivariate ranking and filtering steps. Five different sets of rules were implemented using pragmatic values for the number of features to include (top 50; 50–500), FDR thresholds (0.05 and 0.10) and fold-change cutoff. Thresholds were chosen for convenience rather than clinical significance: FDR50 where features were ranked by significance (FDR from LIMMA), followed by selection of the top 50 features; COMBO0.05 where all features with FDR<0.05 were initially selected, combined with minimum (Simon and Dobbin, 2003) and maximum (500) rules to ensure a minimum but manageable panel size (adding less significant features if fewer than 50, or removing the least significant features if more than 500); FDR0.10.FC0.5 where all features with FDR<0.10 were selected, followed by application of an absolute (log2) fold-change cutoff of 0.5 (requiring ≥0.5 for up- and ≤ −0.5 for downregulated features); FC0.5.TOP50 where all features with an absolute (log2) fold-change cutoff of 0.5 were selected (based on the logFC-value returned by LIMMA), followed by selection of probe-sets with the lowest 50 FDR (accepting less if fewer probe-sets were returned by the FC0.5 selection); FDR0.10.RFE50 where all features with FDR<0.10 were selected, followed by a recursive feature elimination with Support Vector Machines (SVM) analysis and subsequent selection of the 50 features with the largest weight.

For approach FDR0.10.RFE50, the SVM-filtering step represents an additional multivariate ranking and filtering step motivated by recursive-feature-elimination (Guyon et al., 2002) using weights (coefficients) from a linear-kernel SVM (with parameters: cost = 1, type = “C-classification”, kernel = “linear” and scale = TRUE). This step was performed after the univariate filter. A simple, one-time reduction of the number of probe-sets to 50 was performed by this filter using SVM-coefficients returned by the *e1071*-package in R (Meyer et al., 2019). This approach will return 50 features, except when there are fewer than 50 input features with FDR<0.10, and in that case all of them will be returned.

A fold-change filter was implemented where separate upper and lower thresholds were applied to only include features with fold-changes equal to or above the upper threshold, or equal to or below the lower threshold. For the analysis, thresholds were provided on the log2-scale (a threshold of 1 corresponds to a fold-change of 2 for instance). This filter was used for the FDR.0.10.FC0.5 approach as described above.

Classification: To develop parsimonious classifiers for BCAR, a computational pipeline was applied (Günther et al., 2012). Five different uni- and multivariate ranking and filter combinations were used as described above, after which 8 different multivariate classification methods were explored based on six different algorithms: Elastic Net (EN; three variations corresponding to different α parameters covering a range from Lasso to Ridge regression), Linear discriminant analysis (LDA), Shrunken Centroids (PAM), Random Forest (RF), Support Vector Machines (SVM) and Extreme Gradient Boosting (XGBoost) (Friedman et al., 2010; Tibshirani et al., 2002; Breiman, 2001; Chen and Guestrin, 2016; Meyer et al., 2019; Hastie et al., 2009). Multiple k-fold cross-validation runs were performed for parameter tuning and model selection for each of the classification methods. EN and PAM classification methods performed additional feature selection while SVM, RF and XGBoost used the full list of input features from the previous step. LDA by itself does not perform feature selection, but the number of top *n* significant features was used as a tuning parameter.

Parameter tuning: Most classification methods display a range of performance over parameter space, and parameters were tuned to help select best-performing models. A two-step process was applied where an initial analysis explored model performance over a wide parameter range before zooming in on a specific range for the main analysis. All classification models except RF were tuned. A single parameter was used for tuning and additional parameters were fixed. RF was run with all fixed parameters after initial exploration showed no clear dependence of performance on any of *ntree*, *nodesize* and *mtry* parameters. Additional information on parameter tuning is provided in the Supplements.

Model assessment: Parameter tuning and model selection required model assessment which was based on a performance metric and selection criteria. In this study, area under the receiver operating characteristics curve (AUC), classification error, sensitivity and specificity were explored as metrics with either extreme value selection (minimum or maximum) by itself, or in combination with a one standard error (SE) rule, for example, selecting the model with the lowest classification error within one SE of the minimum error (Hastie et al., 2009). Sensitivity and specificity calculations used a classification score (probability of AR) threshold of 0.5.

Eight-fold cross-validation was selected to permit balanced stratification of the 48 analysis samples while preserving the distribution of early and late rejection events across folds. Each fold contained six samples (three BCAR and three NR), enabling consistent representation of clinical timing within training and test sets. Sensitivity and specificity were calculated using a probability threshold of 0.5, reflecting the balanced study design and an assumption of symmetric costs for false-positive and false-negative errors. Threshold optimization was not the objective of this analysis, which focused on comparative evaluation of modeling strategies rather than application-specific decision tuning.

Models were assessed for specific model parameter ranges over multiple cross-validation partitions for tuning, and over multiple nested cross-validation partitions for unbiased performance estimation of the selected model. AUC as estimated by this process was used to characterize expected classification performance in unknown test data (Varma and Simon, 2006).

As an alternative to potentially time-consuming nested cross-validation, a bias correction method for classification error was implemented and explored (Ryan and Robert, 2009). The method was adjusted and also applied to AUC, sensitivity and specificity.

Performance validation: A cross-validation framework was implemented to maximize use of available samples as compared to a hold-out data set approach where training sets are typically smaller. Multiple partition nested cross-validation (MPnCV) was used for unbiased estimation of classifier performance. For consistent use of the same pipeline implementation, the nested CV approach was also applied to the RF classifier which did not require parameter tuning. In nested CV, there is an outer and an inner fold. This is in contrast to regular CV where only one fold-partition is used. When models were tuned, inner fold CV was used to determine best model parameters for different performance measures and criteria. Performance from the outer fold CV was then used to characterize classifier performance. For the main analysis and results, maximum AUC with the one-standard-error rule was used consistently for tuning and model selection.

Performance in cross-validation was determined by testing trained models on samples in the left-out folds, followed by calculation of the mean and standard error of the chosen performance metric over all folds. For single-partition *k*-fold CV, *k* performances were averaged. When *m* partitions were used in multi-partition CV, k*m performances were averaged. Additional details can be found in the Supplementary Material.

Biological analysis: Each of the 200 method combinations (5 pre-filter times 5 uni-/multivariate ranking and filtering methods times 8 classification methods) resulted in one classifier and an underlying probe-set panel. A summary table was created with fold-change, direction, mean expression levels in BCAR and NR groups, as well as a feature inclusion matrix for all probe-sets identified over the 200 panels. Features were mapped to genes with annotation version 36 for Affymetrix HG-U133 Plus2 GeneChips and ordered by how often they were encountered across panels. Additional analyses using the same approach were performed for subsets of the 200 models, focusing on specific pre-filters and classification methods.

Biological pathway analysis: The R package *ReactomePA* was utilized for a pathway enrichment analysis (Yu and He, 2016). The analysis was performed separately for each set of pre-filtered genes with FDR <0.1. This threshold allowed a larger number of candidate genes to be used in the analysis since some of the pre-filtered lists returned as little as 19 probe-sets for FDR<0.05. To assess the significance of enriched pathways, *ReactomePA* applies a hypergeometric strategy, considering the total number of genes in the pathway and the total number of genes in the dataset. Multiple testing correction was performed using Benjamini-Hochberg. Pathway enrichment results were visualized using the *dotplot* function of *ReactomePA* as well as the *emapplot* function of the *enrichplot* package.

Classifier comparison: Each classifier was trained and instructed to return a probability-of-BCAR prediction rather than a classifier-specific score (e.g., LDA score). The probability measure allowed comparison of different classifiers on the same scale. A detailed comparison was based on individual classifier probabilities for all BCAR and NR samples as determined by cross-validation. Probabilities were predicted for different combinations of pre-filter, ranking and filtering and classifications methods and displayed together across all samples in heatmaps and boxplots to allow identification of combinations with outlier characteristics or other distinguishing patterns through visual inspection.

Density distribution, scatter plots and correlations between AUC, cvError, sensitivity and specificity were summarized and inspected for patterns across all method combinations. In addition, a similar analysis was performed for the subset of method combinations which included feature-selecting classifier methods (LDA, PAM and EN). For this analysis, the number of features in the selected classifier model was included.

AUC represents a measure of classification performance across different probability thresholds. It allows systematic comparison of classifiers, and bar graphs of AUC as derived with cross-validation were created for all method combinations together with AUC differences (flat vs. nested). Scatter plots of nested vs. flat CV results for AUC, cvError, sensitivity and specificity were inspected to look for pre-filter and classifier-specific patterns.

Additional information on the analytical procedures is provided in Supplementary Material.

## Results

### Subjects were representative of the total provincial transplant population

A total of 48 subjects were included. Group 1 (cases) comprised 24 subjects with Banff category 2 BCAR (histological grade: Ia = 9, Ib = 3, IIa = 11, III = 1) and Group 2 (disease controls) comprised 24 matched subjects with no clinical or histological rejection. Consistent with the full population of the British Columbia transplant program, patients were predominantly Caucasian, male and received a graft from either living or deceased donors (Table 1). BCAR occurred within the first 6 months post-transplant (mean 19 ± 29 days).

**TABLE 1:** Demographic and clinical characteristics of study subjects. Demographic and clinical characteristics of study subjects. Numbers in brackets are percentages unless otherwise stated.

Characteristics	Acute rejection	No acute rejection
Subjects	24	24
Mean age (s.d.)	47 (11)	47 (11)
Male	17 (71%)	14 (58%)
Ethnicity		
Caucasian	22 (92%)	19 (79%)
Asian Indian	2 (8%)	1 (4%)
Asian Oriental	0 (0%)	2 (8%)
Other	0 (0%)	2 (8%)
Primary disease		
Glomerulonephritis	9 (37%)	10 (42%)
Polycystic kidney disease	1 (4%)	5 (21%)
Diabetic nephropathy	3 (13%)	2 (8%)
Other	11 (46%)	7 (29%)
Donor type		
Living donor	15 (63%)	17 (71%)
Deceased donor	9 (37%)	7 (29%)
Estimated GFR (ml/min/1.73 m^2^)		
Week 1 post Tx	22 ± 13	47 ± 16
Month 3 post Tx	42 ± 14	57 ± 17
Month 12 post Tx	42 ± 14	57 ± 17

### Pre-filtering strategies influenced the number and overlap of retained probe-sets

**FIGURE 1:**
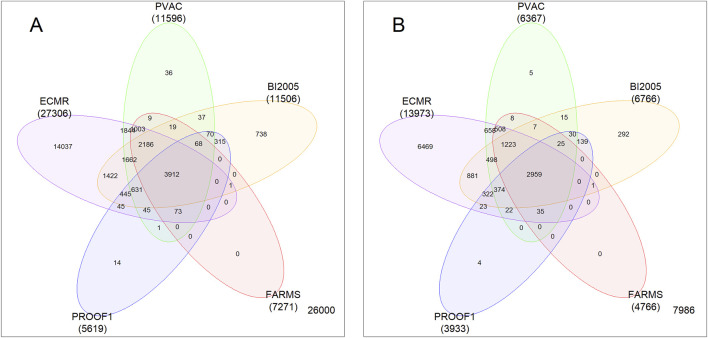
Pre-filter panel comparison. 5-set Venn diagram showing the number and overlap of probe-sets **(A)** and corresponding genes **(B)** on the Affymetrix HG-U133 Plus2 GeneChip that passed each of the 5 pre-filtering methods. Probe-set/gene total count for each method is shown in brackets and is also summarized in Table 2 together with total overlap. The number in the lower right shows the number of probe-sets **(A)** or genes **(B)** on the Affymetrix GeneChip that were not included in any of the pre-filters.

The numbers of probe-sets returned by the five pre-filtering methods are compared in Figure 1 and summarized in Table 2. The most permissive strategy (ECMR) returned 27,306 probe-sets, while the most restrictive (PROOF1) returned fewer than 25% of this number (5,619) with 5,151 probe-sets in common. A total of 11,356 probe-sets were shared between the two most liberal individual pre-filters (ECMR and PVAC), with 98% of PVAC probe-sets also identified by ECMR. In contrast, only 4,053 probe-sets were shared between the two most restrictive methods (FARMS and PROOF1), with 99% and 92% of probe-sets, respectively, also identified by ECMR. The union of all probe-sets was 28,613 while the intersection was 3,912. Overall, 26,000 of 54,613 candidate probe-sets (48%) on the Affymetrix GeneChip were excluded by all pre-filtering strategies. Venn diagrams and expression distributions for all pre-filter combinations are shown in Supplementary Figure S1.

**TABLE 2:** Influence of individual pre-filtering strategies on number of probe-sets differentially expressed in acute rejection (FDR = 0.1), including direction of change, and number of probe-sets for refined false discovery rates (FDR = 0.05 and FDR = 0.01). Probe-set to gene mapping based on NetAffy annotations (v36; 2016).

Pre-filter	ECMR	PVAC	BI2005	FARMS	PROOF1
Total - Probe-sets - Genes	27,306 13,973	11,596 6,367	11,506 6,766	7,271 4,766	5,619 3,933
Unique (not found elsewhere) - Probe-sets - Genes	14,037 6,469	36 5	738 292	0 0	14 4
Differential expression - Probe-sets - Genes	1,042 800	202 153	341 266	173 154	77 72
Direction of change (probe-sets) - Up - Down	576 466	121 81	216 125	108 65	53 24
False discovery rate (probe-sets) −0.05 −0.01	446 73	98 33	134 35	80 22	19 8
Probe-set overlap (total) - ECMR - PVAC - BI2005 - FARMS - PROOF1	11,356 10,259 7,175 5,151	11,356 8,585 7,270 4,800	10,259 8,585 6,186 5,441	7,175 7,270 6,186 4,053	5,151 4,800 5,441 4,053
Gene overlap (total) - ECMR - PVAC - BI2005 - FARMS - PROOF1	6,277 6,258 4,726 3,735	6,277 5,131 4,765 3,445	6,258 5,131 4,215 3,849	4,726 4,765 4,215 3,019	3,735 3,445 3,849 3,019

### Differential expression varied substantially with the pre-filter stringency

The ECMR filter identified 1,042 probe-sets representing 800 defined genes that were differentially expressed between cases and controls (FDR <0.10). Of these, 466 were downregulated and 576 upregulated (FC range: −2.4 to +2.9); 387 had an absolute FC > 1.41 and 31 an absolute FC >2.0, 446 had an FDR <0.05 and 73 an FDR <0.01. At the other extreme, PROOF1 identified 77 probe-sets corresponding to 72 genes differentially expressed between cases and controls (FDR<0.10), including 24 down- and 53 upregulated probe-sets (FC range: −1.93 to +2.35); 19 had an FDR <0.05 and 8 an FDR <0.01. All but one PROOF1 probe-sets were included among those identified by ECMR. The other pre-filters yielded intermediate results (Table 2).

**FIGURE 2:**
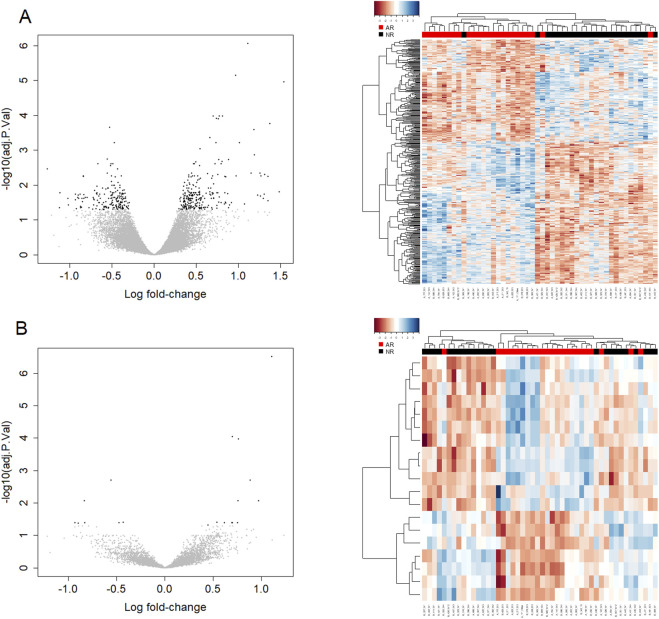
Differential expression analysis for ECMR and PROOF1 pre-filter. Differential expression of probe-sets between subjects with and without BCAR detected by micro-array analysis. **(A)** Analysis of 27,306 probe-sets returned by the EMCR pre-filter and 446 probe-sets identified as significant by LIMMA using a cutoff of FDR = 0.05. **(B)** Analysis of the 5,619 probe-sets returned by the PROOF1 pre-filter and the 19 probe-sets identified as significant by LIMMA using a cutoff of FDR = 0.05. Volcano diagrams show fold change and significance. Points in black on the volcano diagram indicate the probe-sets identified as significant by LIMMA using a cutoff of FDR <0.05. Points in grey represent the remaining probe-sets that passed the pre-filter. Hierarchical cluster analysis shows differentially expressed probe-sets where each column represents one patient sample, each row indicates a probe-set, and the color in each cell represents row-standardized log2-gene expression values; red being low and blue high. The analysis used distance measures ‘euclidean’ for columns and Pearson correlation for rows, and hierarchical clustering method ‘complete’ to determine the dendrograms.

Figure 2 shows volcano plots and heat maps for ECMR (A) and PROOF1 (B) at FDR<0.05. Unsupervised two-way hierarchical clustering showed separation between patients with or without BCAR which was more pronounced for the 446 ECMR probe-sets. Results for all five pre-filters are provided in Supplementary Figure S2.

Results of the most permissive pre-filtering method (ECMR) are shown in Table 3. Five ranking and filtering strategies were applied to 27,306 probe-sets returned by ECMR: (a) COMBO0.05: all probe-sets with an FDR <0.05 (n = 446) with enforced minimum and maximum panel sizes of 50 and 500, (b) TOP50: the top 50 probe-sets with an FDR <0.05, (c) FDR0.10-FC0.5: all probe-sets with FDR0.10 and an absolute FC >1.41 (n = 387); (d) FC0.5-TOP50, selecting the top 50 probe-sets with an absolute FC >1.41, and (e) FDR0.10.RFE50; the top 50 probe-sets with an FDR <0.05 based on calculated weights from the SVM method.

**TABLE 3:** Number of features (probe-sets) and performance of classifier panels according to ranking strategy and multivariate classifier when applied to the 27,306 probe-sets returned by the ECMR pre-filter. For A, performance estimates were derived from outer 8-fold CV over 5-partitions which was used for parameter tuning and final model selection, while for B, estimates are based on 5-partition nested 8-fold CV.

A	Multivariate classifiers								ProbeSet
	EN-0.10	EN-0.50	EN-0.90	LDA	PAM	RF-1	SVM	XGBoost	Mean
FDR<0.05 [50–500]; 446 probe-sets									
Features	10	37	19	9	4	446	446	446	177
AUC	0.9444	0.9694	0.9750	0.9806	0.9306	0.9222	0.9278	0.9333	0.9479
Misclassification	0.1500	0.0667	0.0667	0.0708	0.1542	0.1250	0.1208	0.1333	0.1109
Sensitivity	0.8250	0.8750	0.8750	0.9000	0.8083	0.8333	0.8333	0.8583	0.8510
Specificity	0.8750	0.9917	0.9917	0.9583	0.8833	0.9167	0.9250	0.8750	0.9271
Top50 FDR probe-sets									
Features	10	8	9	9	4	50	50	50	24
AUC	0.9444	0.9472	0.9528	0.9806	0.9333	0.9389	0.9472	0.9250	0.9462
Misclassification	0.1500	0.1417	0.1125	0.0708	0.1458	0.1375	0.1125	0.1458	0.1271
Sensitivity	0.8250	0.8333	0.8583	0.9000	0.8167	0.8500	0.8583	0.8417	0.8479
Specificity	0.8750	0.8833	0.9167	0.9583	0.8917	0.8750	0.9167	0.8667	0.8979
FC >1.41; FDR<0.1; 372 probe-sets									
Features	10	9	6	9	3	372	372	372	144
AUC	0.9500	0.9694	0.9694	0.9806	0.9250	0.9167	0.9278	0.9472	0.9483
Misclassification	0.1375	0.1292	0.1125	0.0750	0.1667	0.1500	0.1250	0.1000	0.1245
Sensitivity	0.8417	0.8417	0.8500	0.9000	0.7917	0.8333	0.8167	0.8500	0.8406
Specificity	0.8833	0.9000	0.9250	0.9500	0.8750	0.8667	0.9333	0.9500	0.9104
FC > 1.41; Top50 probe-sets									
Features	10	9	6	9	4	50	50	50	24
AUC	0.9500	0.9694	0.9694	0.9806	0.9361	0.9472	0.9500	0.9389	0.9552
Misclassification	0.1375	0.1292	0.1125	0.0750	0.1583	0.1375	0.1083	0.1167	0.1219
Sensitivity	0.8417	0.8417	0.8500	0.9000	0.8000	0.8500	0.8583	0.8667	0.8510
Specificity	0.8833	0.9000	0.9250	0.9500	0.8833	0.8750	0.9250	0.9000	0.9052
FDR<0.1; SVM-top50 probe-sets									
Features	21	21	15	7	23	50	50	50	30
AUC	0.9278	0.9444	0.9583	0.9417	0.9333	0.9361	0.9444	0.9056	0.9365
Misclassification	0.0917	0.0833	0.0833	0.1583	0.1167	0.0958	0.1042	0.1250	0.1073
Sensitivity	0.8417	0.8583	0.8583	0.8667	0.8250	0.8250	0.8000	0.8500	0.8406
Specificity	0.9750	0.9750	0.9750	0.8167	0.9417	0.9833	0.9917	0.9000	0.9448
Overall									
Features	12	17	11	9	8	194	194	194	80
AUC	0.9433	0.9600	0.9650	0.9728	0.9317	0.9322	0.9394	0.9300	0.9468
Misclassification	0.1333	0.1100	0.0975	0.0900	0.1483	0.1292	0.1142	0.1242	0.1183
Sensitivity	0.8350	0.8500	0.8583	0.8933	0.8083	0.8383	0.8333	0.8533	0.8463
Specificity	0.8983	0.9300	0.9467	0.9267	0.8950	0.9033	0.9383	0.8983	0.9171
FDR<0.05 [50–500]; 446 probe-sets									
Features	10	37	19	9	4	446	446	446	177
AUC	0.9194	0.9306	0.9333	0.9333	0.9361	0.9222	0.9306	0.9333	0.9299
Misclassification	0.1542	0.1417	0.1333	0.1583	0.1417	0.1250	0.1167	0.1292	0.1375
Sensitivity	0.8333	0.8500	0.8500	0.8333	0.8333	0.8333	0.8333	0.8500	0.8396
Specificity	0.8583	0.8667	0.8833	0.8500	0.8833	0.9167	0.9333	0.8917	0.8854
Top50 FDR probe-sets									
Features	10	8	9	9	4	50	50	50	24
AUC	0.9222	0.9306	0.9333	0.9333	0.9389	0.9389	0.9278	0.9306	0.9319
Misclassification	0.1583	0.1625	0.1458	0.1583	0.1417	0.1375	0.1333	0.1458	0.1479
Sensitivity	0.8250	0.8417	0.8333	0.8333	0.8333	0.8500	0.8417	0.8333	0.8365
Specificity	0.8583	0.8333	0.8750	0.8500	0.8833	0.8750	0.8917	0.8750	0.8677
FC >1.41; FDR<0.1; 372 probe-sets									
Features	10	9	6	9	3	372	372	372	144
AUC	0.9222	0.9417	0.9389	0.9333	0.9278	0.9167	0.9139	0.9083	0.9253
Misclassification	0.1708	0.1542	0.1583	0.1583	0.1625	0.1500	0.1375	0.1208	0.1516
Sensitivity	0.8167	0.8417	0.8500	0.8333	0.8000	0.8333	0.8000	0.8667	0.8302
Specificity	0.8417	0.8500	0.8333	0.8500	0.8750	0.8667	0.9250	0.8917	0.8667
FC >1.41; Top50 probe-sets									
Features	10	9	6	9	4	50	50	50	24
AUC	0.9222	0.9306	0.9417	0.9333	0.9389	0.9472	0.9333	0.9111	0.9323
Misclassification	0.1667	0.1583	0.1458	0.1583	0.1458	0.1375	0.1458	0.1333	0.1490
Sensitivity	0.8167	0.8417	0.8500	0.8333	0.8250	0.8500	0.8333	0.8500	0.8375
Specificity	0.8500	0.8417	0.8583	0.8500	0.8833	0.8750	0.8750	0.8833	0.8646
FDR<0.1; SVM-top50 probe-sets									
Features	21	21	15	7	23	50	50	50	30
AUC	0.9000	0.9139	0.9306	0.9306	0.8972	0.9361	0.9444	0.8944	0.9184
Misclassification	0.1375	0.1458	0.1208	0.1333	0.1833	0.0958	0.1083	0.1542	0.1349
Sensitivity	0.8083	0.8083	0.8333	0.8417	0.7500	0.8250	0.8083	0.8250	0.8125
Specificity	0.9167	0.9000	0.9250	0.8917	0.8833	0.9833	0.9750	0.8667	0.9177
Overall									
Features	12	17	11	9	8	194	194	194	80
AUC	0.9172	0.9294	0.9356	0.9328	0.9278	0.9322	0.9300	0.9156	0.9276
Misclassification	0.1575	0.1525	0.1408	0.1533	0.1550	0.1292	0.1283	0.1367	0.1442
Sensitivity	0.8200	0.8367	0.8433	0.8350	0.8083	0.8383	0.8233	0.8450	0.8313
Specificity	0.8650	0.8583	0.8750	0.8583	0.8817	0.9033	0.9200	0.8817	0.8804

### Classifier performance varied across method combinations and pre-filtering strategies

Forty classifier models were developed by combining the five ranking and filtering strategies COMBO0.05 (FDR <0.05; n = 446; panel size constrained to 50–500), TOP50 (top 50 by FDR <0.05), FDR0.10.FC0.5 (n = 387), FC0.5.TOP50, and FDR0.10.RFE50 (top 50 based on SVM weights) with eight classifiers - Elastic Net (three variants), LDA, PAM, RF, SVM, and XGBoost. Model selection used a 5-partition, 8-fold cross-validation framework, applying a maximum-AUC one–standard error criterion with preference for smaller panels.

**FIGURE 3:**
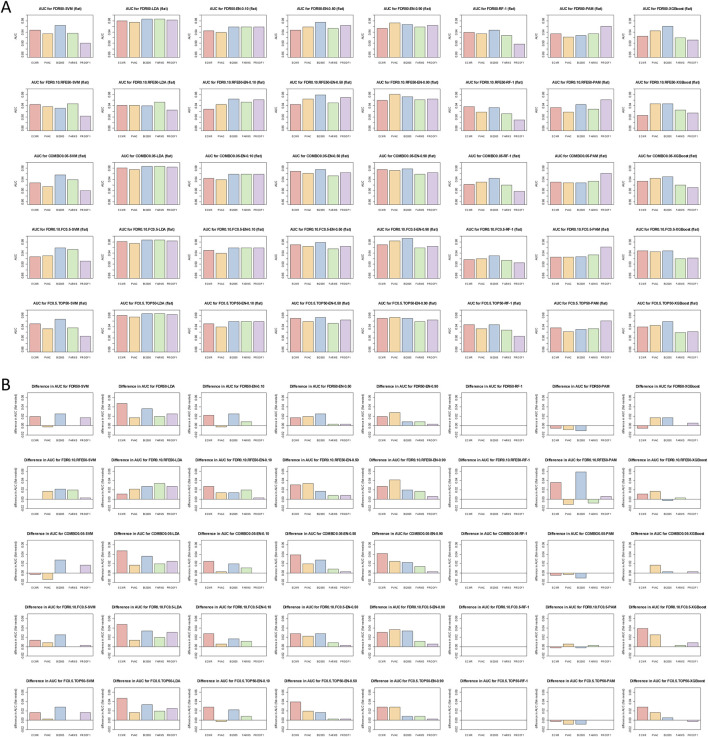
Classifier performance comparison. **(A)** Comparison of performance (AUC) by pre-filter for each of 40 method combinations and outer 8-fold CV over 5 partitions. Colors indicate pre-filter method. AUC-range set to [0.85–1] to highlight differences. **(B)** Difference in performance from flat (biased) and nested (unbiased) CV for the same combinations. No parameters were tuned for random forest and flat and nested CV performance estimates were the same. Supplementary Figure S19 shows ROC-curves corresponding to **(A)**.

Across all five pre-filters, classifier performance was generally high, with most method combinations achieving AUCs above 0.88 (Figure 3A). However, both absolute performance and susceptibility to over-fitting varied systematically with pre-filter stringency and classifier choice (Figure 3B). More permissive pre-filters (ECMR, PVAC) tended to yield higher apparent performance but also greater evidence of over-fitting, particularly for LDA and XGBoost-based models. In contrast, more restrictive pre-filters (FARMS, PROOF1) showed reduced over-fitting at the cost of smaller feature sets and, in some cases, modestly lower AUCs.

PAM and elastic net classifiers demonstrated the most consistent resistance to over-fitting across pre-filters, particularly when combined with intermediate stringency strategies such as BI2005. Elastic net models showed a general trend toward improved discrimination with decreasing pre-filter size, except for BI2005-based models, which consistently outperformed FARMS-based models in the EN-0.5 and EN-0.9 settings. LDA models exhibited the greatest variability, with pronounced over-fitting under ECMR except when paired with FDR0.10.RFE50, and consistently selected very small panels across pre-filters. Overall, low classification error was typically accompanied by higher specificity and lower sensitivity, reflecting conservative classification behavior across methods (Table 3B; Supplementary Figure S3A).

### Biological interpretation of selected features revealed consistent patterns across classifier strategies

**FIGURE 4:**
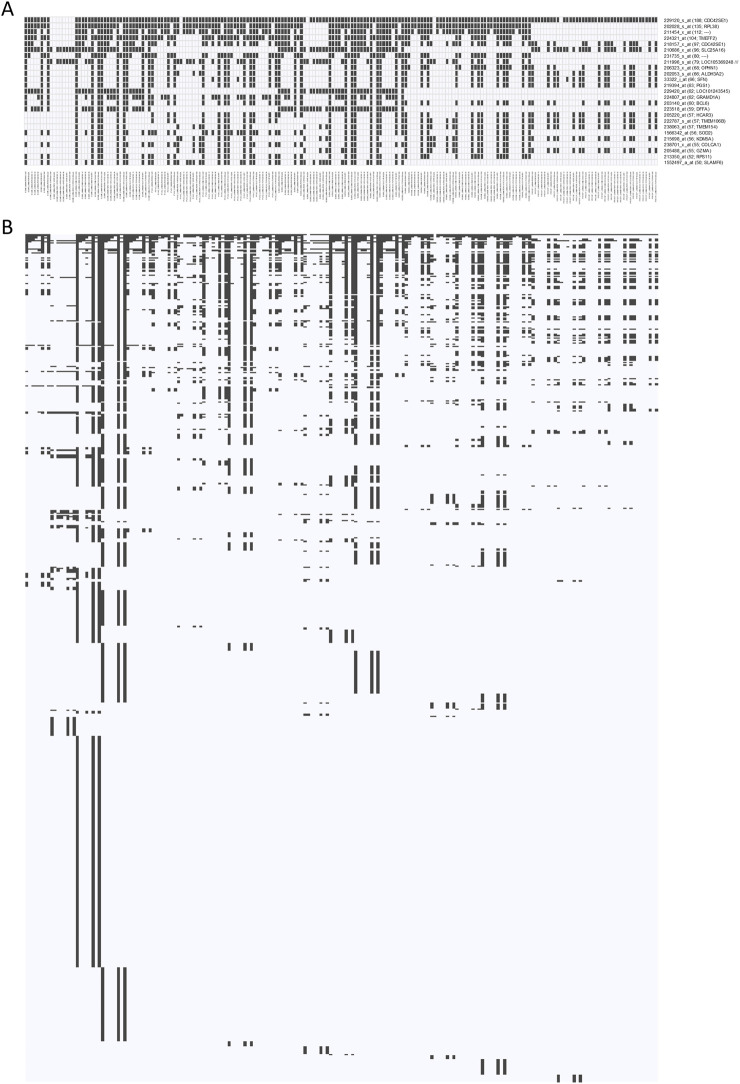
Classifier panel comparison. Binary probe-set inclusion matrix for 200 classifier panels identified in the analysis (columns) and 25 probe-sets that were observed in at least 50 (25%) of the 200 panels **(A)**, and all 688 probe-sets **(B)**.

Table 4 summarizes genes corresponding to the most frequently selected probe-sets over 200 classifier panels. In total, 688 unique probe-sets were observed. Applying a 25% frequency threshold identified 25 probe-sets that appeared in at least 50 panels. Feature inclusion matrices and frequency plots are shown in Figure 4A and Supplementary Figure S11 respectively; results for all 688 probe-sets are shown in Supplementary Table S4. Stratified frequency analyses are shown in Supplementary Figures S12–S14.

**TABLE 4:** Gene summary for the most frequently found probe-sets (≥25%) over 200 classifier panels from five pre-filtering approaches. There were 25 probe-sets identified in the union of 688 probe-sets over 200 panels and the corresponding frequency distribution is shown in Supplementary Figure S11. Observed count and fraction over 200 panels, gene symbol and gene name are included in the table. Probe-set to gene mapping is based on NetAffy annotations (v36; 2016). The complete table with all 688 probe-sets can be found in Supplementary Table S4.

ProbeSet	GeneSymbol	GeneTitle	Mean BCAR	Mean NR	Fold Change	Change in BCAR	Count	Fraction
229120_s_at	CDC42SE1	CDC42 small effector 1	9.43	8.32	2.16	Up	188	0.94
202028_s_at	RPL38	Ribosomal protein L38	8.82	7.85	1.95	Up	135	0.68
211454_x_at	---	---	9.21	8.40	1.75	Up	112	0.56
224321_at	TMEFF2	Transmembrane protein with EGF-like and two follistatin-like domains 2	8.10	6.56	2.91	Up	104	0.52
218157_x_at	CDC42SE1	CDC42 small effector 1	9.50	8.74	1.70	Up	97	0.49
210686_x_at	SLC25A16	“Solute carrier family 25 (mitochondrial carrier), member 16″	9.44	8.74	1.62	Up	96	0.48
231735_s_at	---	---	9.80	8.43	2.59	Up	80	0.40
211996_s_at	LOC105369248///	Nuclear pore complex-interacting protein family member B5-like///…	7.57	6.85	1.65	Up	79	0.40
206323_x_at	OPHN1	Oligophrenin 1	10.07	9.19	1.85	Up	68	0.34
202053_s_at	ALDH3A2	“Aldehyde dehydrogenase 3 family, member A2″	7.05	7.89	1.79	Down	66	0.33
33322_i_at	SFN	Stratifin	6.99	7.55	1.47	Down	66	0.33
219394_at	PGS1	phosphatidylglycerophosphate synthase 1	8.15	7.39	1.69	Up	63	0.32
229420_at	LOC101243545	Uncharacterized LOC101243545	7.11	6.34	1.71	Up	62	0.31
224807_at	GRAMD1A	GRAM domain containing 1A	7.19	6.54	1.58	Up	62	0.31
203140_at	BCL6	B-cell CLL/lymphoma 6	11.22	10.24	1.96	Up	60	0.30
223518_at	DFFA	“DNA fragmentation factor, 45 kDa, alpha polypeptide”	6.55	7.08	1.44	Down	59	0.30
205220_at	HCAR3	hydroxycarboxylic acid receptor 3	10.20	9.44	1.69	Up	57	0.29
222787_s_at	TMEM106B	Transmembrane protein 106B	7.30	8.23	1.91	Down	57	0.29
238063_at	TMEM154	Transmembrane protein 154	9.27	8.65	1.54	Up	57	0.29
1566342_at	SOD2	“Superoxide dismutase 2, mitochondrial”	9.11	7.93	2.27	Up	56	0.28
215698_at	KDM5A	lysine (K)-specific demethylase 5A	7.21	8.15	1.91	Down	56	0.28
238701_x_at	COLCA1	colorectal cancer associated 1	10.58	9.78	1.74	Up	55	0.28
205488_at	GZMA	granzyme A	6.54	7.25	1.64	Down	55	0.28
213350_at	RPS11	Ribosomal protein S11	8.61	7.42	2.28	Up	52	0.26
1552497_a_at	SLAMF6	SLAM family member 6	6.58	7.24	1.58	Down	50	0.25

Among the ten most frequent probe-sets, nine were increased in BCAR, including *CDC42SE1* (2x), *RPL38*, *TMEFF2*, *SLC25A16*, *OPHN1*, *LOC105369248* and two non-annotated probe-sets, while only one, *ALDH3A2*, was decreased. Seven of the top 25 probe-sets were decreased in BCAR, including *ALDH3A2*, *SFN*, *DFFA*, *TMEM106B*, *KDM5A*, *GZMA* and *SLAMF6*.

The FDR - SVM (RFE50) strategy yielded a more heterogeneous profile, preferentially selecting downregulated features (12 of 21 probe-sets among the most frequent). Upregulated genes included *CDC42SE1*, *LOC105369248*, and *SLC25A16* together with *HCG27*, *RPL38*, *RBPJ* and *TMEM154* while downregulated genes comprised *ALS2CR12*, *KDM5A*, *GZMH*, *GZMA*, *KLRC4*-*KLRK1*, *SLAMF6*, *SYTL2*, *LINC00520*, *TGFBR3*, *MFHAS1*, *SNHG17* and *EGFR*. Only 178 of 688 probe-sets observed across all panels were selected in the RFE50 subset.

### Biological pathway analysis identified coherent immune pathways across pre-filtering strategies

**FIGURE 5:**
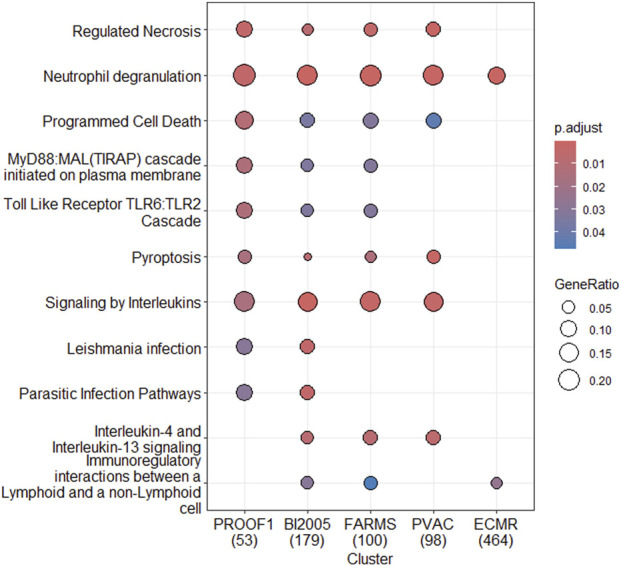
Reactome pathway enrichment analysis. Results of the Reactome pathway enrichment analysis using genes with FDR<0.1 of the five different pre-filters as input. The dotplot visualizes which pathways are enriched when using the different pre-filters. The gene ratio is the number of DE genes mapped to the Reactome pathway in question divided by number of DE genes mapped to any Reactome pathway. The number in brackets on the x-axis is the denominator of the gene ratio.

Pathway enrichment analysis was performed for each pre-filter using the genes differentially expressed at FDR< 0.1. Several pathways were consistently identified across pre-filters (Figure 5) including *neutrophil degranulation* (identified by all five pre-filters), and *regulated necrosis, programmed cell death, pyroptosis,* and *signaling by interleukins* (identified by four of the five). Additional pathways, including *MyD88MAL(TIRAP) cascade, TLR6:TLR2 Cascade, IL-4/IL-13 signalin*g and *immunoregulatory interactions between lymphoid and non-lymphoid cells* were identified by three pre-filters, supporting biological coherence across analytical pipelines.

### Classifier comparison highlighted systematic differences across methods and patient samples

**FIGURE 6:**
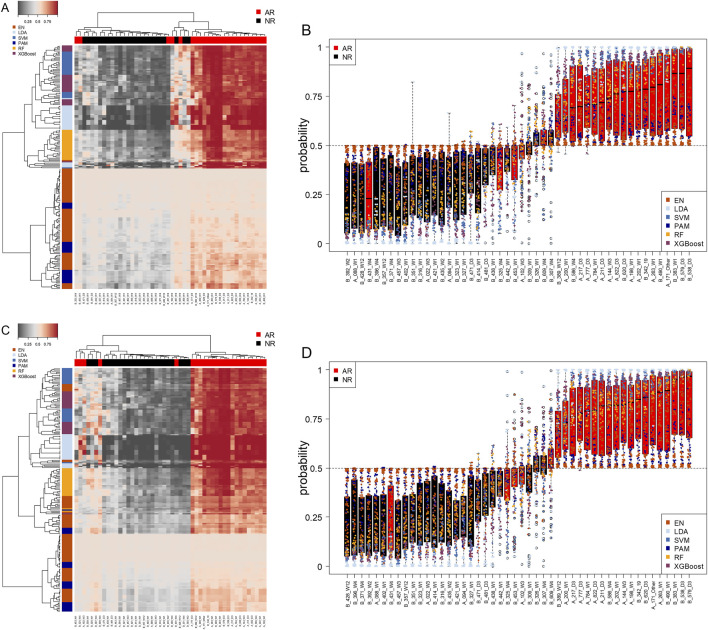
Classification score clustering and comparison. Shown are heatmap **(A,C)** and boxplot **(B,D)** representations of 9,600 classification scores (averaged over 5 cross-validation partitions; top **(A,B)**: nested CV; bottom **(C,D)**: flat CV) for 48 samples, 5 pre-filter methods and 40 classification methods. Scores represent probability of AR with low scores corresponding to high probability of NR. Columns represent individual patient samples, color-coded red for AR and black for NR. Each row represents a different method combination, and row label colors indicate the general type of classification algorithm (EN-Elastic Net, LDA-Linear Discriminant Analysis, SVM-Support Vector Machine, PAM-Shrunken Centroid, RF-Random Forest and XGBoost-Extreme Gradient Boosting). The whiskers in the boxplots **(B,D)** use the default setting in R’s boxplot-function, i.e., they extend to the most extreme data point which is no more than 1.5 times the interquartile range from the box.

Classifier score distributions across patients and methods are shown in Figure 6, enabling direct comparison of method combinations. Heatmaps (Figure 6A) and boxplots (Figure 6B), present the summarized AR probabilities (0–1) for all 48 patients across 200 method combinations (5 pre-filters x 40 classifier configurations) based on maximum AUC one-standard-error selection. Scores representing the probability of acute rejection were estimated using 5-times repeated 8-fold cross validation with nested CV. Figures 6C,D show outer-fold (biased) estimates. Compared to Figure 6B, outlier scores above 0.5 for non-rejecting samples and below 0.5 for rejecting samples were no longer observed. Several AR samples clustered with NR samples and showed probabilities below 0.5. One AR sample consistently exhibited low scores across most classifiers. No NR sample showed a strong AR pattern, although two had median scores slightly above 0.5. Wide inter-quartile ranges reflected variability across classifiers, LDA models produced extreme scores near 0 or 1, largely independent of the pre-filter whereas EN and PAM produced scores closer to 0.5. Increasing EN alpha values were associated with greater AR and NR separation.

Additional data on individual classifier output and performance is provided in the Supplementary Tables and Figures.

## Discussion

High-throughput genomic and proteomic technologies now permit the simultaneous assessment of thousands of molecular features, enabling detailed interrogation of immune activation, tissue injury, and repair processes in transplantation and other complex diseases (Ong and Mann, 2005; Hayden et al., 2003; Henger et al., 2004; Kurian et al., 2007; Bermúdez-Crespo and López, 2007; Mirza and Olivier, 2008; Simon and Dobbin, 2003; Irizarry et al., 2003; Smyth et al., 2005; Gentleman et al., 2004). In kidney transplantation, both tissue-based biomarker panels and individual molecular markers identified within the allograft have improved diagnostic precision, prognostication, and therapeutic stratification in acute rejection (Sarwal et al., 2003; Einecke et al., 2008; Famulski et al., 2007; Mueller et al., 2007). However, repeated tissue sampling is invasive and clinically impractical, motivating increasing interest in minimally invasive sources such as peripheral blood to capture biologically meaningful signals associated with rejection and graft injury (Aquino-Dias et al., 2008; Marrer and Dieterle, 2007; Flechner et al., 2004; Brouard et al., 2007). Within this context, convergent functional genomics has emerged as an important framework for integrating high-dimensional molecular data with biological interpretation (Kurian et al., 2007; Le-Niculescu et al., 2008).

In the present study, acute renal allograft rejection served as a practical model to examine how analytical choices at different stages of a transcriptomic pipeline, specifically pre-filtering, feature ranking and selection, and classifier modeling, affect classification performance, robustness, and biomarker refinement. Using a balanced cohort of 48 patients (24 with BCAR and 24 non-rejecting controls), we systematically evaluated 200 analytical pipelines. Given the modest sample size, the primary aim was not to establish a definitive diagnostic signature for acute rejection, but rather to explore how methodological variation influences downstream results in a biologically meaningful yet analytically controlled setting.

Across all method combinations, substantial variability was observed in classification performance metrics, including AUC, classification error, sensitivity, and specificity. Importantly, no single analytical strategy consistently outperformed others across all conditions. At the same time, all evaluated pipelines achieved estimated AUCs ≥0.88, suggesting that multiple methodological routes can recover informative signals from the data. Taken together, these results indicate that apparent differences in classifier performance are often driven more by analytical design choices than by fundamental biological differences in the underlying signal.

A wide range of supervised learning and regularization approaches was examined, although many additional methods are available in the broader machine learning literature (Pargent et al., 2022; Cerulli and Cerullil, 2023). More classical regularized models and feature-selecting classifiers remain well suited to modestly sized transcriptomic data sets (Zhao et al., 2024; Zhao et al., 2025a). Recent advances in artificial intelligence and deep learning have yielded impressive results in large-scale biomedical applications, particularly in image analysis and multi-omic integration (Zhao et al., 2022; Zhao et al., 2025b). In addition to supervised classifiers, unsupervised techniques such as self-organizing maps, t-SNEand robust PCA have been widely used for structure discovery and dimensionality reduction in high-dimensional molecular data, alongside an emerging set of AI-based classification approaches (Kohonen, 1989; van der Maaten and Hinton, 2008; Cai et al., 2021; Munquad et al., 2022; Mahin et al., 2022). Deep learning methods typically require substantially larger training cohorts than were available in this study and are known to perform unreliably in small-sample settings (LeCun et al., 2015; Ching et al., 2018) although these techniques have been applied to cancer studies using microarray data and might prove beneficial in acute kidney rejection studies as well (Gupta et al., 2022; Xiao et al., 2018).

Pre-filtering emerged as a central determinant of downstream analytical behavior. The methods evaluated differed substantially in their underlying objectives, emphasizing noise reduction (BI2005), variance filtering (ECMR), or probe consistency (PVAC and FARMS). These differences resulted in nearly five-fold variation in the number of retained probe-sets, with direct consequences for feature availability, classifier stability, and susceptibility to over-fitting. No single pre-filtering strategy emerged as uniformly optimal. More permissive approaches increased the likelihood of retaining biologically relevant features but also elevated the risk of introducing irrelevant markers into classifier panels. Conversely, more stringent pre-filters reduced over-fitting but at the cost of feature diversity and, in some cases, classification flexibility.

These findings suggest that pre-filtering should be viewed not as a purely technical preprocessing step, but as a critical design decision that shapes the analytical search space. Confidence in candidate biomarkers was greatest when probe-sets passed multiple complementary pre-filters, those enforcing probe consistency alongside variance and signal thresholds, for example, supporting the use of combined or consensus pre-filtering strategies in biomarker discovery workflows. Such approaches may be particularly valuable in settings where biological heterogeneity and technical noise coexist.

Among the classification methods examined, elastic net and PAM classifiers demonstrated a favorable balance between discrimination performance, robustness, and parsimony. Given the generally high performance observed across pipelines, smaller feature panels are particularly attractive from both interpretability and translational perspectives. Elastic net models with higher α values and PAM classifiers achieved strong discrimination with fewer than 20 genes, highlighting their suitability for developing clinically tractable assays. By contrast, LDA tended to exhibit more pronounced over-fitting under permissive pre-filters, reflecting sensitivity to feature ordering and tuning strategy, while XGBoost performance was highly dependent on pre-filter stringency and feature composition.

The comparative behavior of classification algorithms observed here is consistent with prior work in other high-dimensional molecular settings, where regularized linear models such as elastic net and margin-based methods such as support vector machines have frequently demonstrated strong and stable performance relative to alternative approaches (Zhuang et al., 2012). More broadly, our analytical strategy deliberately emphasized comparison across multiple method combinations to clarify how pre-filtering, univariate and multivariate feature selection, and classifier choice jointly shape model performance and robustness. In contrast to many biomarker studies that evaluate only a limited number of analytical pipelines, this approach aligns with a growing body of benchmark and comparative investigations demonstrating that no single feature-selection or modeling strategy is uniformly optimal across datasets or endpoints (Li et al., 2022; Mahendran et al., 2020; Baali et al., 2024; Qin et al., 2022; Hamla et al., 2023; Zucknick et al., 2008; Theng and Bhoyar, 2024). Regardless of classifier choice, however, rigorous validation remains central to reliable performance assessment in high-dimensional transcriptomic studies, particularly in small-sample settings where overfitting and optimistic bias are well recognized risks (Boulesteix et al., 2008; Barbash and Soreq, 2013; Sánchez-Maroño et al., 2019; Lever et al., 2016).

Probability-based analyses provided additional insight into classifier behavior across methods. Heatmap and boxplot visualizations enabled comparison of predicted probabilities across classifiers and samples, revealing subsets of patients with highly consistent predictions, others with borderline probabilities near 0.5, and a small number of samples that appeared consistently discordant. Taken together, these patterns illustrate the value of ensemble-based perspectives, not only for performance estimation but also for quality control, annotation review, and identification of clinically ambiguous cases. Rather than relying on a single classifier output, such visualizations can help contextualize uncertainty and guide downstream clinical interpretation.

Despite the primary methodological emphasis of this study, the biological coherence of the identified signals was notable. Pathway enrichment analysis (Jassal et al., 2020) consistently highlighted immune and inflammatory processes across multiple pre-filtering strategies, including *neutrophil degranulation, regulated necrosis, programmed cell death, pyroptosis, cytokine signaling, and TLR–MyD88* pathways. These pathways are well established in the pathophysiology of acute allograft injury and rejection. Neutrophils are among the earliest immune cells to infiltrate transplanted organs and contribute to ischemia–reperfusion injury and early inflammatory signaling (Qu et al., 2023; Scozzi et al., 2017). Similarly, *regulated necrosis* and *pyroptosis* have increasingly been recognized as contributors to graft injury and immune activation in rejection (Sarhan et al., 2018), (Linkermann, 2016). The consistent identification of interleukin signaling and TLR pathways further supports the biological plausibility of the transcriptomic signatures detected across diverse analytical pipelines (Zhang et al., 2017) (Braza et al., 2016; Sharbafi et al., 2019).

## Limitations

Several limitations should be acknowledged. Whole blood was selected as the sampling matrix to avoid artefacts introduced by cell separation; however, this approach cannot distinguish gene expression changes driven by altered cell composition from those reflecting altered cellular function. Although differential expression was observed specifically at the time of BCAR, suggesting biological robustness, cell-type–specific effects and compositional shifts warrant further investigation using complementary approaches. Our new studies will therefore employ single-cell methods to disentangle cell-specific and compositional effects.

Strict phenotypic selection limited sample size and precluded evaluation under more complex clinical conditions, such as delayed graft function or concurrent infection. The distribution of borderline cases may reflect heterogeneous biology or reduced sensitivity of blood-based profiling relative to tissue expression. While nested cross-validation was employed to mitigate optimistic bias, performance estimates were derived from models trained on fewer samples than the final classifiers, and we acknowledge the importance of independent data sets to determine unbiased estimates of classifier performance (Vabalas et al., 2019; Aliferis et al., 2024; Krstajic et al., 2014).

Probabilities were constrained to the [0,1] interval to facilitate comparison across classifiers. However, score distributions differed between methods and should be considered when interpreting ensemble results. Small sample size also limited model training in some cross-validation folds, particularly for LDA-based approaches, and such incomplete models should be discounted when comparing performance.

## Conclusions, implications and future directions

This study shows that transcriptomic biomarker discovery in transplantation is strongly influenced by analytical design choices, particularly pre-filtering strategy and classifier selection. The computational pipeline began with 54,613 variables, and substantial differences were observed in the number of retained variables following pre-filtering. While liberal and stringent pre-filters produced markedly different candidate feature spaces, no single analytical pipeline consistently outperformed others. Instead, robust classification performance emerged across a range of method combinations, emphasizing that reproducibility and convergence across approaches are more informative than reliance on any single model or feature set.

Importantly, intermediate pre-filtering strategies combined with feature-selecting classifiers, particularly elastic net and PAM, offered a favorable balance between discrimination performance, robustness, and parsimony. These approaches consistently yielded small, interpretable biomarker panels with strong estimated performance, supporting their suitability for translational assay development. The absence of a large, universally shared probe-set panel across pipelines further highlights the importance of analytical redundancy and cross-method validation in high-dimensional biomarker studies.

From a practical perspective, these findings suggest that method selection should be guided by study objectives rather than maximal apparent performance alone. Application-specific considerations, including tolerance for false positives versus false negatives, stability of classifier scores, and feasibility of panel implementation, should inform analytical choices. When computational or sample resources are limited, evaluation of at least two complementary classifiers can substantially increase confidence in identified signals (Naidu et al., 2023).

Looking forward, validation in independent, multicenter cohorts will be essential to determine the generalizability of candidate biomarker panels. Future studies incorporating longitudinal sampling, larger and more diverse populations, and multi-omic integration are likely to further refine these approaches. More broadly, these findings support the pursuit of transcriptomic biomarker discovery as a comparative and iterative process, integrating methodological rigor with biological plausibility to advance clinically meaningful precision diagnostics in transplantation.

## Data Availability

The original contributions presented in the study are included in the article/Supplementary Material, further inquiries can be directed to the corresponding author.
