# Supplementary material for: Bioinformatics strategies and biomarker refinement using high-throughput transcriptome data in transplantation
Source: Front Bioinform. 2026 Apr 8;6:1677453. doi: 10.3389/fbinf.2026.1677453 (PMC13099910; doi:10.3389/fbinf.2026.1677453)

Supplementary Information and Materials

Contents

Microarray normalization 2
R, Bioconductor and R-package versions 2
ECMR pre-filter 2
Ranking and Filtering Method Overview 3
Description of Performance Calculation 4
Parameter tuning 6
Practical considerations 7
References 8
Supplementary Figure 1 9
Supplementary Figure 2 10
Supplementary Figure 3 11
Supplementary Figure 4 12
Supplementary Figure 5 13
Supplementary Figure 6 14
Supplementary Figure 7 15
Supplementary Figure 8 16
Supplementary Figure 9 17
Supplementary Figure 10 18
Supplementary Figure 11 19
Supplementary Figure 12 20
Supplementary Figure 13 21
Supplementary Figure 14 22
Supplementary Figure 15 23
Supplementary Figure 16 24
Supplementary Figure 17 25
Supplementary Figure 18 26
Supplementary Figure 19 27
Supplementary Figure 20 28
Supplementary Figure 21 29
Supplementary Figure 22 30

Microarray normalization

The Microarray data processing involved three steps known as the RMA-method [1]: (1) Background correction: A probabilistic model is used to background-correct probe-level data independently for each microarray, (2) Quantile normalization: Background corrected probe-level data on each microarray is normalized to a common set of quantiles, derived from the background-corrected data from all microarrays, and (3) Expression calculation: Expression values are estimated separately for each probe-set using median polish. We have used the RMA-normalization as provided in the RefPlus-package version 1.13.2 (2009-03-11) on Bioconductor 2.7 to normalize a set of 293 Microarray CEL files (on 2010-12-10), including multi time-point samples for 62 kidney transplant patients [2,3]. We used 48 of those 293 samples for analysis, one sample per patient (24 AR and 24 NR) with description in the text.

R, Bioconductor and R-package versions

The analysis was performed with MRAN-4.0.2 and Bioconductor version 3.12, using the previously normalized Microarray data for 48 samples. The classification analysis used R-packages: MASS version 7.3-53 for LDA, e1071 version 1.7-3 for SVM, glmnet version 4.0-2 for Elastic Net (EN), pamr version 1.56.1 for Shrunken Centroids (PAM), randomForest version 4.6-14 for Random Forest (RF) and xgboost version 1.2.0.1 for extreme gradient boosting (XGBOOST).

ECMR pre-filter

The Empirical Central Mass Range (ECMR) is defined in Eq.(1) where f_1 is the fraction of the smallest class, e.g. $f_1 = min\left(\frac{N_{AR}}{N_{AR}+N_{NR}}, \frac{N_{NR}}{N_{AR}+N_{NR}}\right)$ in the 2-class classification problem of acute renal allograft rejection. In this quantile-based pre-filter, all probe-sets are ranked according to ECMR and only those probe-sets with values above the median ECMR are filtered. This removes probe sets with little variation across analysis samples, independent of sample class. By using the median, the approach removes half of the 54,613 probe-sets on the Affymetrix Human Genome U133 Plus 2 GeneChip.

$$ECMR(x) = quantile\left(x, 1 - \frac{f_1}{2}\right) - quantile\left(x, \frac{f_1}{2}\right) \quad (1)$$

For balanced class sizes, as is the case in our study, f1 equals 0.5 and ECMR is the same as the inter-quartile range (IQR). For unbalanced class sizes, the ECMR definition in Eq.(1) allows variation in the smaller class to enter the calculation of the quantile range. This avoids scenarios where the smaller group could be left out in the top or bottom quarter of the data, in which case their presence in the data could be missed by the filter. The ECMR-approach drops a fraction of the smallest values of the quantile range without worrying about dropping probe-sets that are very extreme in the smaller group compared to the larger as those would be good biomarkers that would not pass the pre-filtering stage.

Ranking and Filtering Method Overview

The following five ranking and filtering methods were used for the analysis:

- **FDR50**: Rank features by significance (LIMMA; adj.p.Val), and select the Top50.
- **FDR0.10.RFE50**: Select all features with adj.p.Val.<0.10, then run SVM (with cost=1, type="C-classification", kernel="linear", scale=TRUE) and select 50 features with the highest weight.
- **COMBO0.05**: Select all features with adj.p.Val.<0.05; ensure a minimum of 50 & maximum of 500 features (i.e. add 'less' significant features or remove 'least' significant features to stay within a preferred panel size).
- **FDR0.10.FC0.5**: Select all features with adj.p.Val.<0.10 & apply an absolute (log2) fold-change cutoff of 0.5 (so require ≥0.5 for up- and ≤ -0.5 for down-regulated features). Note that the fold-change is calculated from the data and does not use LIMMA's logFC.
- **FC0.5.TOP50**: Select all features with an absolute (log2) fold-change cutoff of 0.5 based on the logFC-value returned by LIMMA, then select the top 50. If there are less than 50 features, use that list, otherwise use the top 50.

Description of Performance Calculation

AUC, misclassification error, sensitivity and specificity were used to characterize model performance. For model selection, additional criteria were used, e.g. smallest model for which performance is within one-standard error of the maximum (for AUC) or minimum (for misclassification error) value. For the multi-partition and nested cross-validation approach, calculation of performance can be done in different ways.

The implementation that produced the analysis results used a multi-partition approach within the inner loop of the nested cross-validation to mimic the model-building process exactly and match the multi-partition approach in the outer loop. A schematic diagram of the outer loop is shown in Supplementary Figure 21. We used 8-fold cross-validation of the balanced data set of 24 acute rejection (AR) and 24 non-rejection (NR) samples. This resulted in 6 samples per fold (3AR and 3 NR). For each outer loop, we trained a classification model on seven folds and were able to use 8 times 6 samples in the left-out folds for testing, either for parameter tuning of the final model, or for estimating classification performance for models built in the inner loop.

We used a multi-partition approach where we ran cross-validation analysis five times with different random partitions of the 48 samples. We therefore had 5 times 8 folds with 6 samples each available for testing and our performance calculation was based on averaging performance over the individual performances from the 40-folds. For our AUC calculation for example, we calculated 40 AUCs, one per fold - based on 6 samples - and averaged to get the AUC values reported in Table 3 (for the ECMR prefilter example). We used the same procedure of averaging over 40 folds to estimate classification error, sensitivity and specificity (using a score threshold of 0.5 where score was probability-of-AR).

For estimation of classification performance of the final model, we used a multi-partition, nested cross-validation approach, where, for each outer loop in Supplementary Figure 21, we set up an inner cross-validation loop as shown in Supplementary Figure 22. We mimic the outer loop cross-validation in the nested loops, so within each outer loop, we also used 5 partitions with 7-folds each. This design resulted in 5*8*5*7=1,400 inner folds.

For classification model building (tuning) and final model performance estimation, the following high-level steps were applied:

1. Start with pre-filtered data set
2. Setup multi-partition, nested cross-validation:
    a. Create 5 partitions with 8-folds each (Supplementary Figure 21).
    b. For each outer test-fold, create 5 partitions with 7 folds each (Supplementary Figure 22).
3. Run LIMMA (univariate analysis; differential expression analysis)
4. Apply univariate, multivariate and fold-change filters to select features for use in classifier training
5. Train classifiers for different parameters (tuning) and test in left-out folds to select models
6. Develop final model using outer cross-validation folds for parameter tuning
7. Use nested loops to develop models for performance estimation in outer loops
    a. Determine performance by averaging selected performance criteria over 40 outer folds

For Supplementary Figure 19 and Supplementary Figure 20, a modified approach was applied where probability-of-AR scores for 240 samples from 5 times 8 test folds were used to determine ROC-curves. This allowed displaying one ROC-curve per model combination, and five ROC-curves for five pre-filter methods in one figure, similar to the AUC bar graphs in Figure 3A. Otherwise, each ROC-curve would need to be replaced by 40 individual curves, each of which would only be based on 6 samples. The two approaches produce slightly different AUC values.

We use the ECMR-FDR50-SVM example to provide a description of the classifier development and performance calculation steps using the multi-partition, nested cross-validation approach. This is one of 200 method combinations we looked at (5 pre-filter methods, 5 uni- and multi-variate ranking and filtering methods and 8 classification methods). The ECMR-FDR50-SVM combination uses the ECMR prefilter which returns 27,306 features, the FDR50 univariate-ranking method which selected the 50 features with the lowest FDR as returned by LIMMA, and the Support Vector Machine classifier algorithm (SVM) with linear kernel and cost as a tuning parameter.

Algorithm for ECMR-FDR50-SVM example

- `For (outer_partition in op1, op2, op3, op4, op5) do`
    - `For (outer_loop in 1, 2, 3, 4, 5, 6, 7, 8) do`
        - `Run LIMMA (42 samples in outer loops)`
        - `For (inner_partition in ip1, ip2, ip3, ip4, ip5) do`
            - `For (inner_loop in 1, 2, 3, 4, 5, 6, 7) do`
                - `Run LIMMA (36 samples in inner loops)`
                - `Apply univariate filter (FDR50)`
                - `Train SVM models for a range of cost parameters`
                    - `(0.0001,0.001,0.01,0.1,1,10,100,1000)`
            - `End (inner_loop)`
        - `End (inner_partition loop)`
        - `Calculate average AUC and standard error over all 5*7=35 inner test folds for all parameter values`
        - `Select the smallest cost parameter (SVM model) where AUC is within one standard error of the maximum average AUC`
        - `Train SVM model with the selected cost parameter on all 7 folds of the current outer_loop`
    - `End (outer_loop)`
- `End (outer_partition loop)`
- `Calculate average AUC over all 5*8=40 outer test folds to estimate performance of the final ECMR-FDR50-SVM model.`

Parameter tuning

Most classification methods display a range of performance over parameter space, and parameters were tuned to help select best-performing models. A two-step process was applied where an initial analysis explored model performance over a wide parameter range before zooming in on a specific range for the main analysis. All classification models were tuned, except RF which was run with all fixed parameters after initial exploration showed no clear dependence of performance on any of *ntree*, *nodesize* and *mtry*.

A single parameter was used for tuning each method and additional parameters were fixed. Tuning more than one parameter requires a more complicated tuning setup where each additional parameter is estimated with an additional nested loop in cross-validation, or where the multi-parameter space is converted to a single dimension. Both approaches have drawbacks, but R-packages exist that could be explored for multi-parameter tuning, e.g. the caret-package which uses a grid search approach [4].

Parameter tuning was done by varying a single parameter for each classification method, followed by calculation of performance metrics AUC, classification error, sensitivity and specificity over multiple k-fold cross-validation, and applying either extreme value selection (minimum or maximum) by itself, or in combination with a one standard error (SE) rule to select the best model, e.g. selecting the model with the lowest classification error within one SE of the minimum error.

For LDA, the number of top *n* significant features was used as a tuning parameter. Some of the method packages, for example *glmnet* for Elastic Net, provide internal cross-validation for parameter tuning. This functionality was not used. Instead, cross-validation for all methods was performed with the inner-outer loop cross-validation as described in the text, and schematically shown in the graphical abstract.

Practical considerations

After pre-filtering, uni- and multi-variate ranking and filtering steps are applied to help focus on a subset of relevant features for classifier training. Depending on the filter settings, it is possible that for a given data set, too few markers are available, and one might observe zero panel sizes for some of the cross-validation partitions. This should be checked, and if observed, is a potential sign of not having a large enough sample size for the respective pre-filter. In this case, one should try to reduce stringency requirements or switch to using a more liberal pre-filter.

Over-fitting can happen when sample size is too small, and the model learned specific rather than general aspects of the data when the best performing model is chosen in the parameter tuning & model selection step. Using the within-one-standard-error approach can guard against over-fitting by selecting a less complex model with less opportunity to memorize the data. A counter-effect to this is that MPnCV runs are based on a smaller sample size with models being trained on 42 samples which in general is expected to have lower performance, so even if there was no over-fitting taking place, performance as determined in the MPnCV runs would be expected to be somewhat lower when sample size is already small. This effect should become less pronounced as sample size increases.

References

1. Irizarry RA, Hobbs B, Collin F, al. et. Exploration, normalization, and summaries of high density oligonucleotide array probe level data. Biostatistics. 2003;4: 249–64.

2. Harbron C, Chang K-M, South MC. RefPlus: an R package extending the RMA Algorithm. Bioinformatics. 2007;23: 2493–2494. doi:10.1093/bioinformatics/btm357

3. RefPlus. In: Bioconductor [Internet]. [cited 29 Oct 2024]. Available: http://bioconductor.org/packages/RefPlus/

4. Kuhn [aut M, cre, Wing J, Weston S, Williams A, Keefer C, et al. caret: Classification and Regression Training. 2024. Available: https://cran.r-project.org/web/packages/caret/index.html

Notes[1].

[1] The RefPlus-package was removed with Bioconductor 3.20 release and is not available anymore.

Supplementary Figure 1

Supplementary Figure 1. Pairwise pre-filter comparison. Pairwise Venn diagrams for the 5 pre-filtering methods showing number and overlap of probe-sets on the Affymetrix HG-U133 Plus2 GeneChip that passed the respective pre-filter method. Diagonal panels show the distribution of gene expression values for 48 samples over all probe-sets that passed the respective pre-filter. In the panels displaying Venn diagrams, probe-set total count for each method is shown in brackets. Numbers in the lower right show the number of probe-sets on the Affymetrix GeneChip that were not included in either of the two pre-filters.

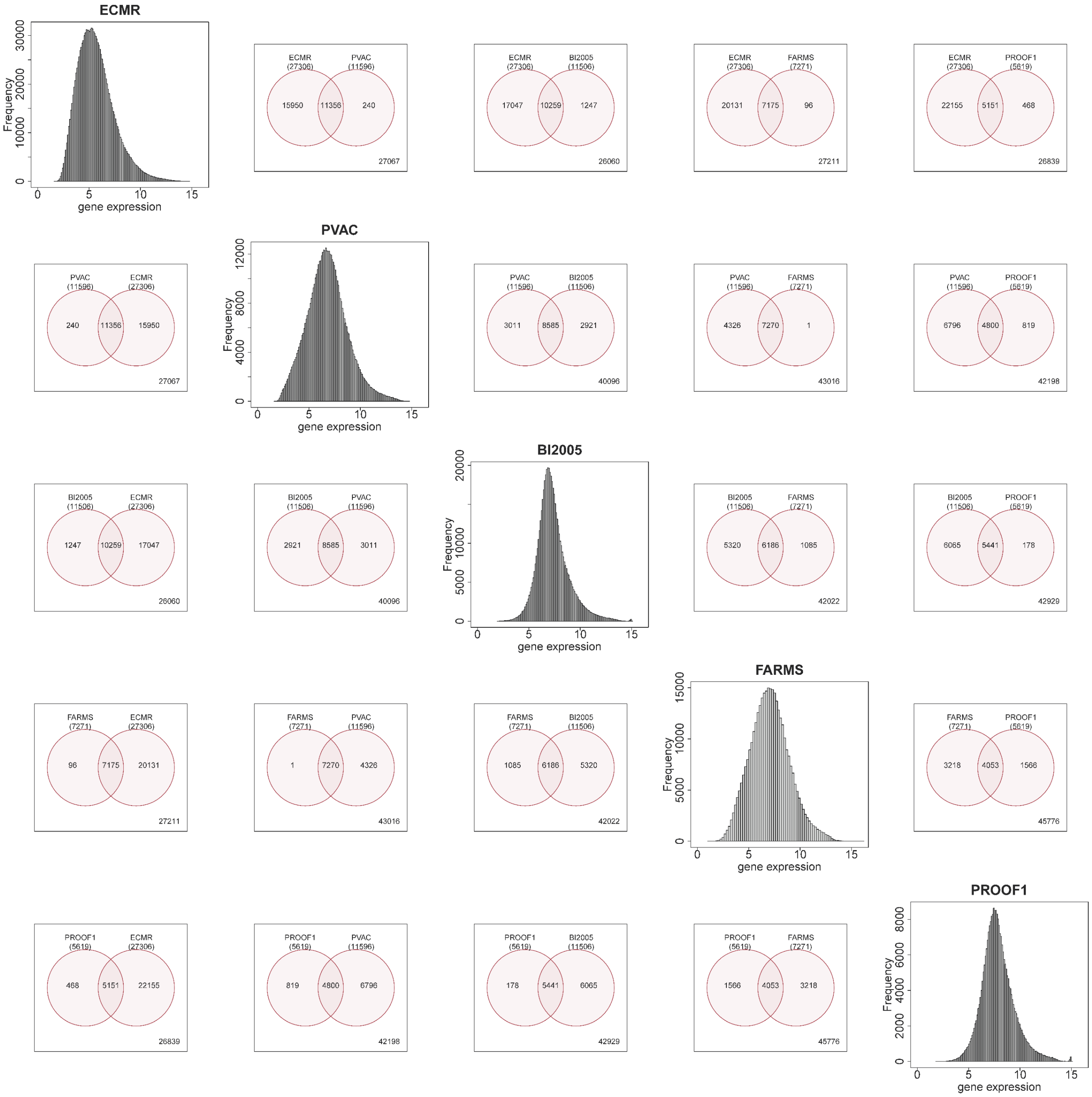

Supplementary Figure 2

Supplementary Figure 2. Differential expression analysis for all pre-filters. Differential expression of probe-sets between subjects with and without BPAR detected by micro-array analysis. Volcano diagrams in the top row show fold change and significance. Points in black on the volcano diagram indicate the probe-sets identified as significant by LIMMA using a cutoff of FDR < 0.05. Points in grey represent the remaining probe-sets that passed the pre-filter. Hierarchical cluster analysis shows differentially expressed probe-sets where each column represents one patient sample, each row indicates a probe-set, and the color in each cell represents row-standardized log2-gene expression values; red being low and blue high. The analysis used distance measures 'euclidean' for columns and Pearson correlation for rows, and hierarchical clustering method 'complete' to determine the dendrograms.

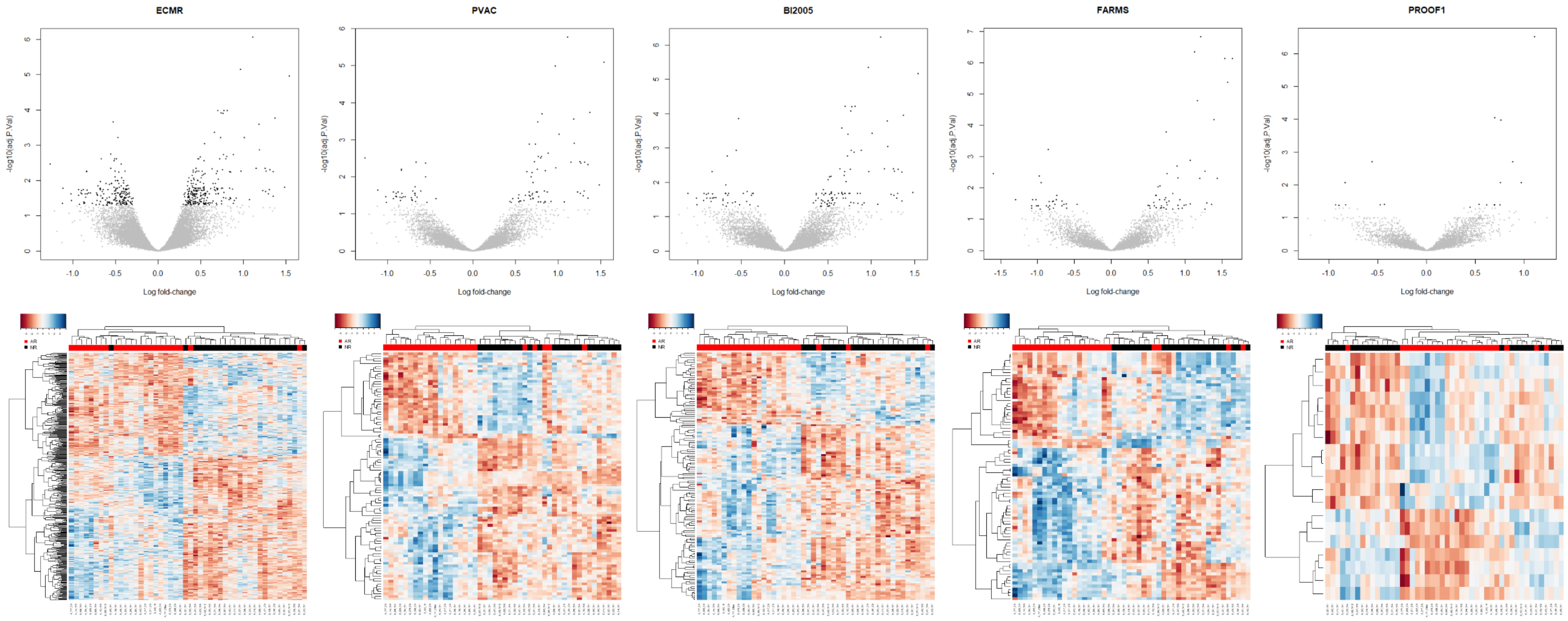

Supplementary Figure 3

Supplementary Figure 3. Classifier performance comparison (nested CV). Comparison of performance (AUC) by pre-filter for each of 40 method combinations and nested 8-fold CV over 5 partitions. Colors indicate pre-filter method. AUC-range set to [0.85-1] to highlight differences. The plot complements Figure 3 which shows similar results based on outer 8-fold CV (flat CV) as well as the difference (flat-nested) to characterize over-fitting and bias. Supplementary Figure S20 shows the corresponding ROC-curves.

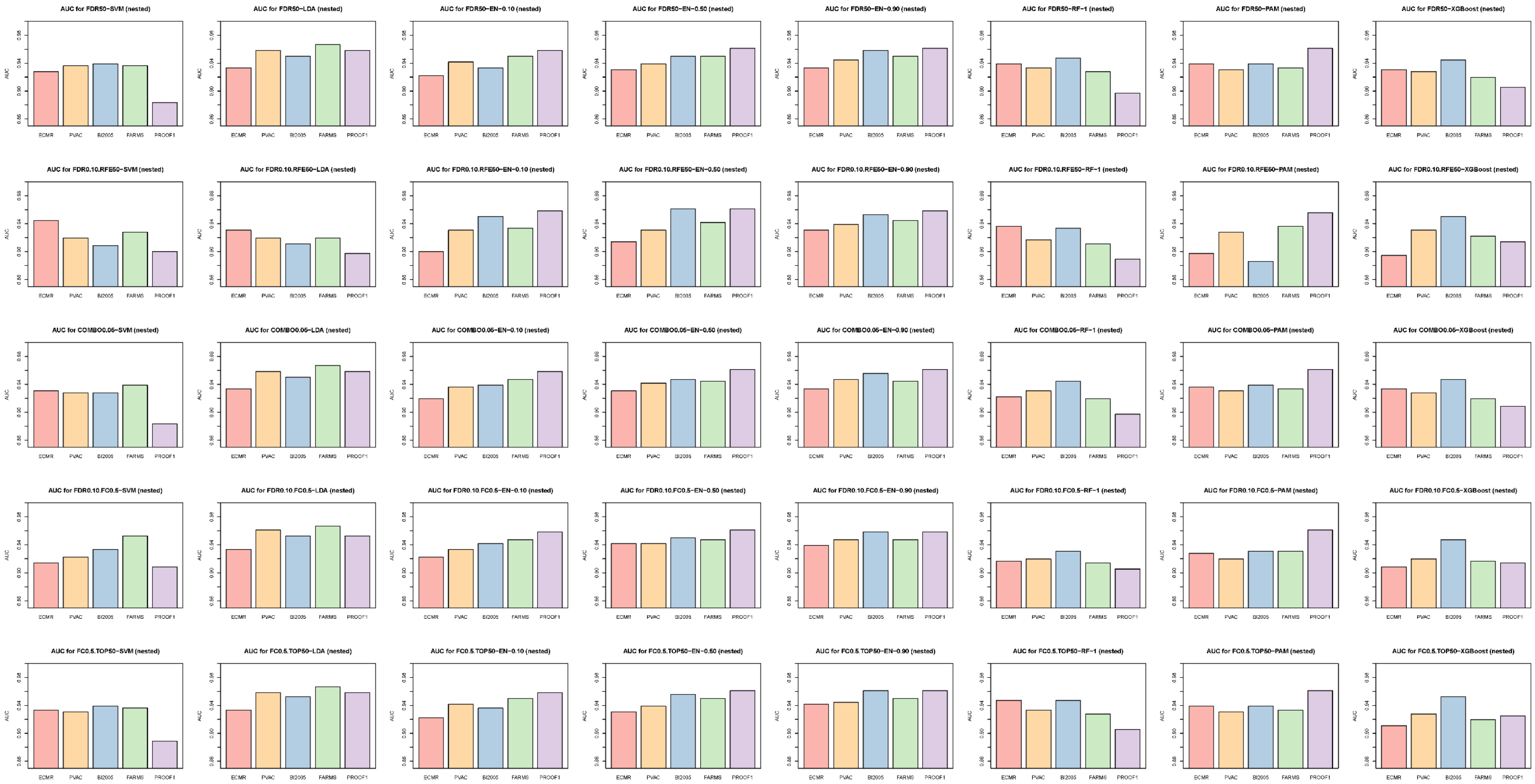

Supplementary Figure 4

Supplementary Figure 4. Performance Scatter (nested vs flat-bias-corrected; derived from AUC_one_se_max). Shown are scatter plot for AUC, cvError, sensitivity and specificity nested vs flat-bias-corrected CV, colored by pre-filter (top row) and classifier (bottom row). Parameter tuning and model selection was based on a maximum AUC within one SE criteria. The jitter function is used to slightly jitter the points to display combinations with the same value pairs.

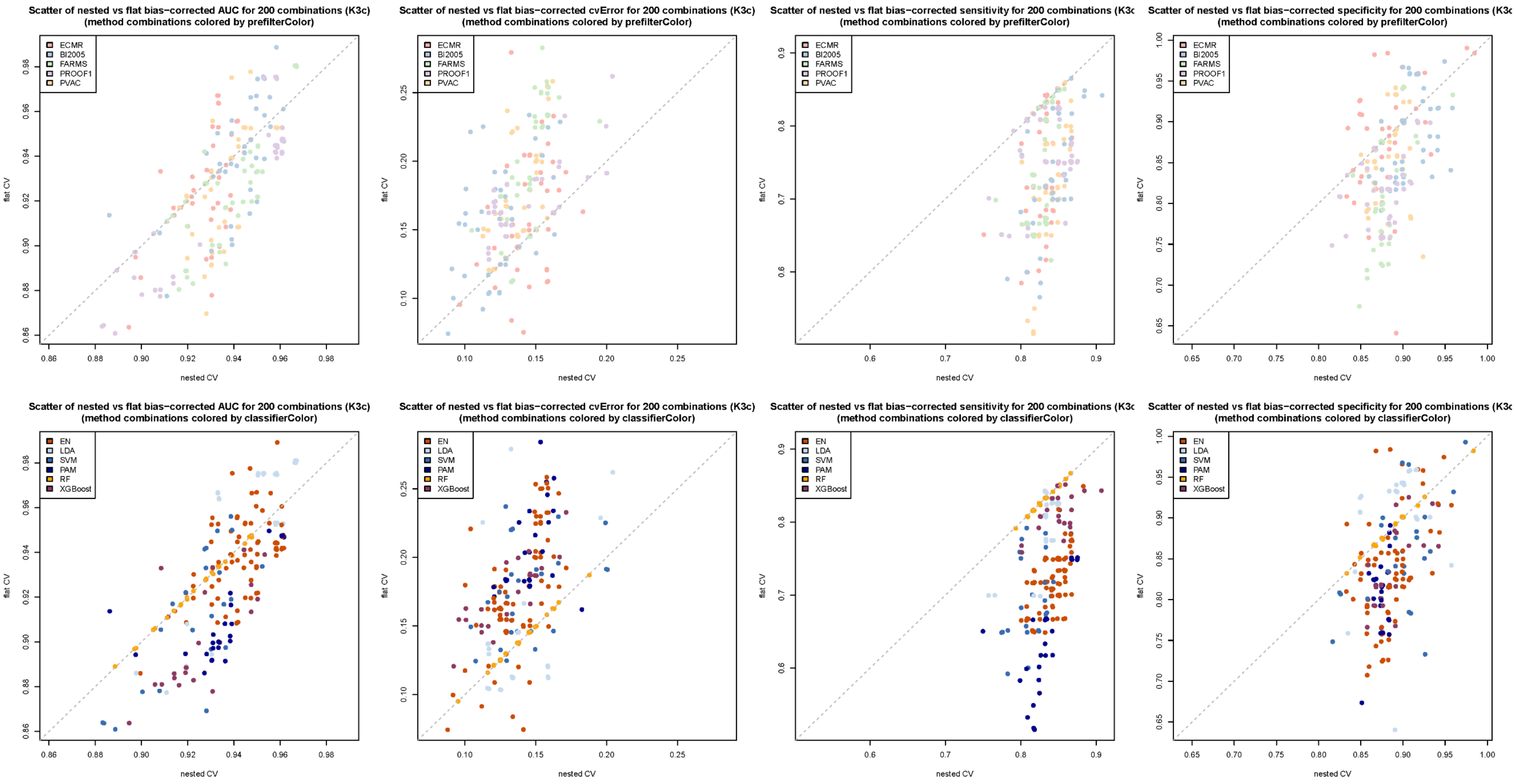

Supplementary Figure 5

Supplementary Figure 5. Performance Scatter (nested vs flat-bias-corrected; derived from cvError_min). Shown are scatter plot for AUC, cvError, sensitivity and specificity nested vs flat-bias-corrected CV, colored by pre-filter (top row) and classifier (bottom row). Parameter tuning and model selection was based on a minimum cvError criteria. The jitter function is used to slightly jitter the points to display combinations with the same value pairs.

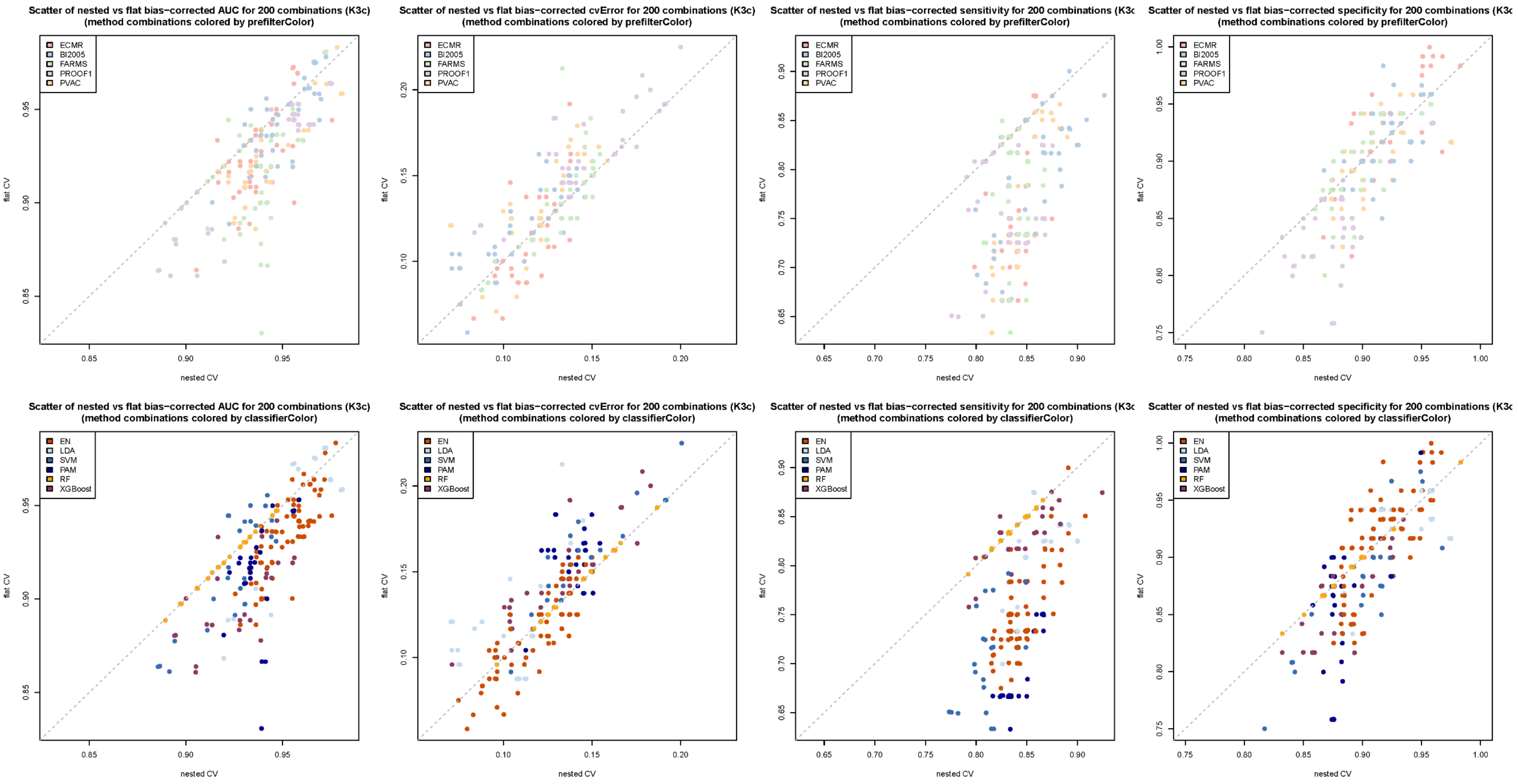

Supplementary Figure 6

Supplementary Figure 6. Parameter tuning and model selection (ECMR; AUC-one-SE-max). Summary of parameter tuning and model selection for 40 method combinations based on ECMR pre-filtered data. The AUC-one-SE-max selection rule was applied which resulted in selection of the parameter shown by the red circle. No parameter was tuned for random forest (RF).

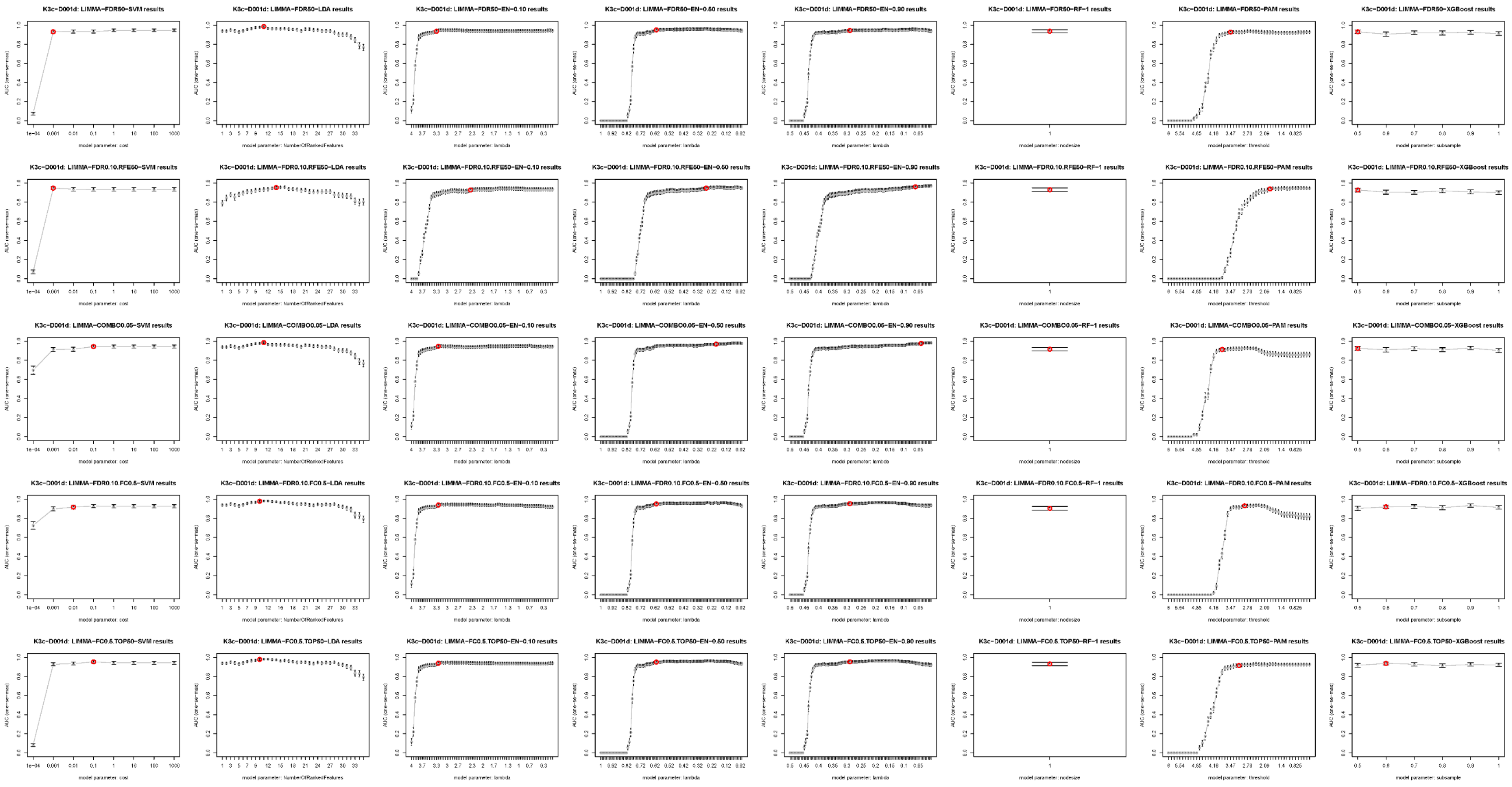

Supplementary Figure 7

Supplementary Figure 7. Probability-of-AR distributions (ECMR; AUC-one-SE-max). Probability-of-AR plots from outer-fold CV for 40 method combinations for the ECMR pre-filter data. The underlying model parameters were selected with AUC and the one standard error within maximum rule, and they are given in the respective panel plot title.

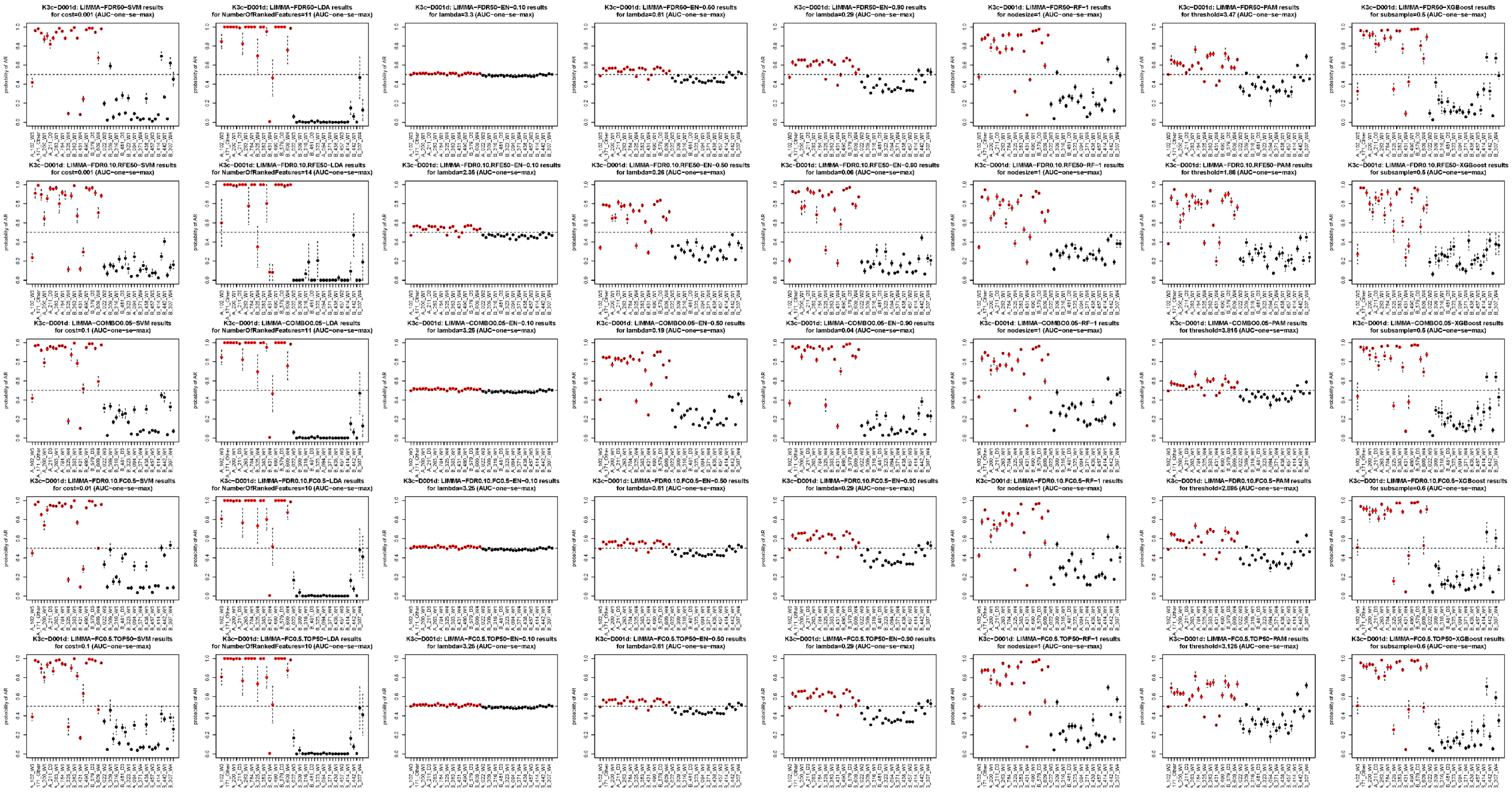

Supplementary Figure 8

Supplementary Figure 8. Impact of model selection criteria. Impact of performance metric on parameter tuning and corresponding model size for two examples for the ECMR pre-filter. Results for FDR50 and EN0.10 in the top row show how model selection can be quite different when using AUC (red circle) vs misclassification error (blue circle), while for COMBO0.05 and PAM in the bottom row, there is little difference. The one-standard error selection rule was used in all cases.

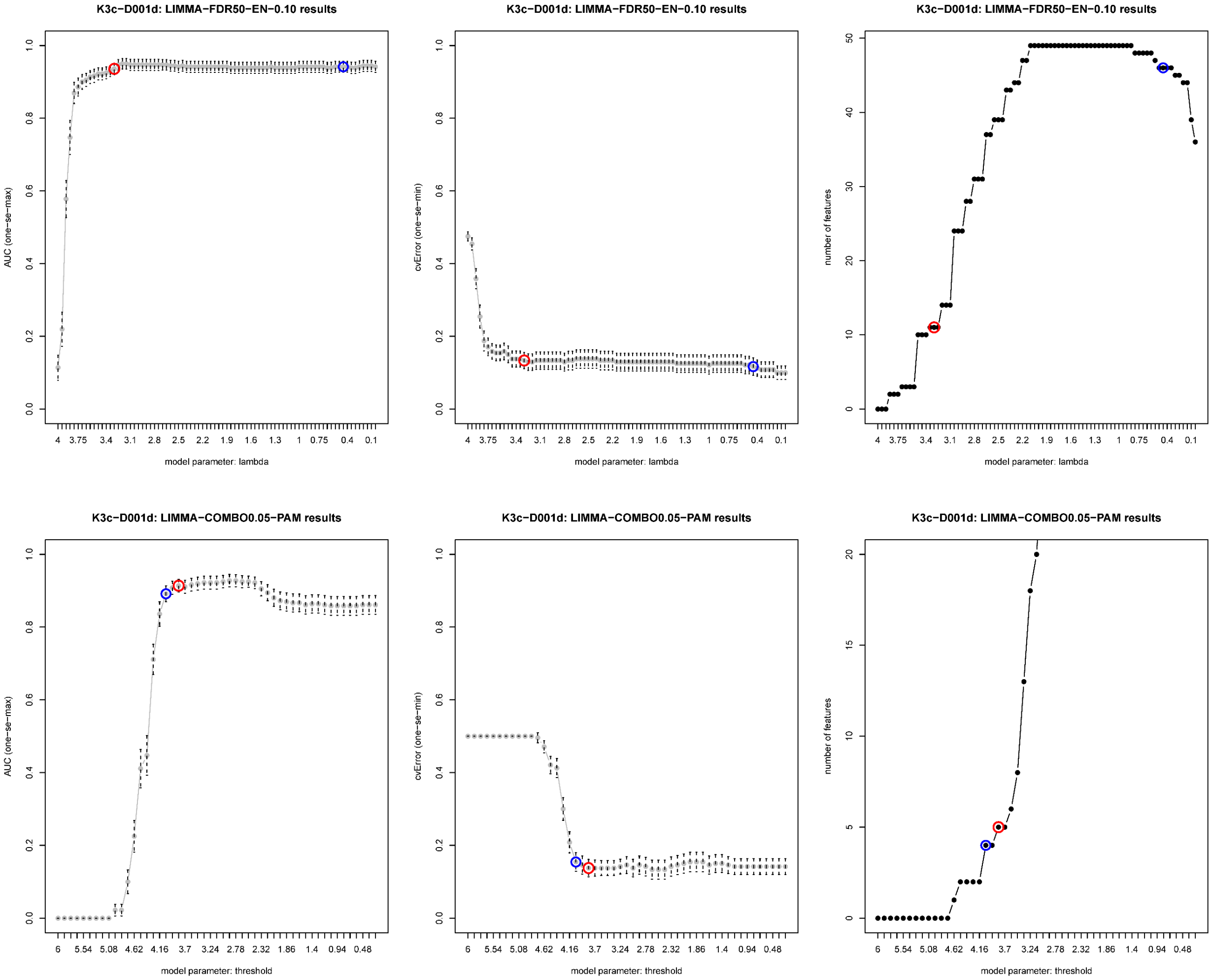

Supplementary Figure 9

Supplementary Figure 9. Probability-of-AR distributions (ECMR; cvError-one-SE-min). Probability of AR plots from multi-partition, outer-fold CV for 40 method combinations for the ECMR pre-filter data. The underlying model parameters were selected with cross-validation error and the one standard error within minimum rule, and they are given in the respective panel plot title.

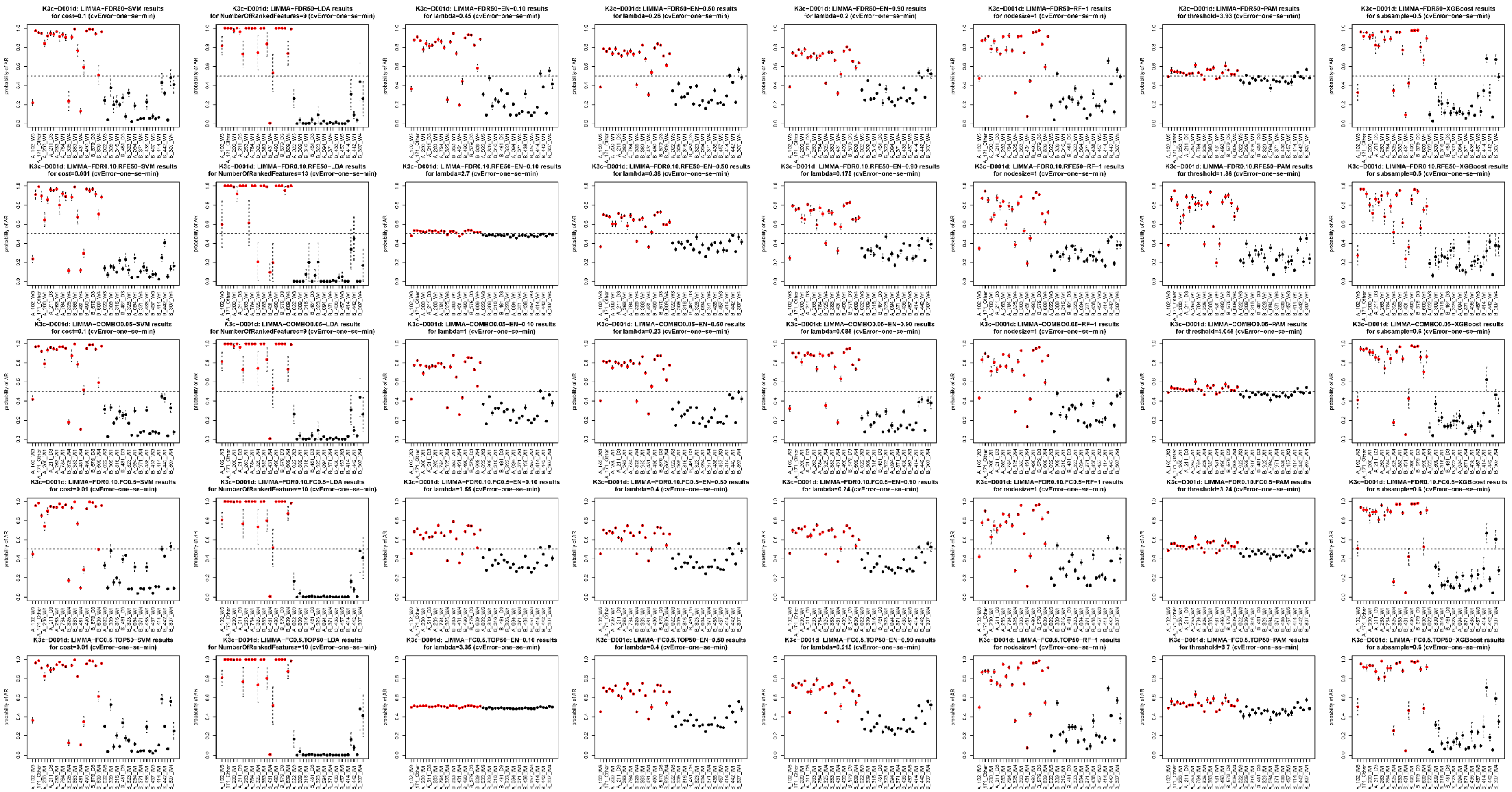

Supplementary Figure 10

Supplementary Figure 10. Classification score clustering for different model selection rules. Heatmaps show classification scores for the FDR50 ranking/filtering method for models selected with flat CV using AUC-one-SE-max (A) or cvError-one-SE-min (B) model selection criteria. Rows show 40 method combinations (5 pre-filter; 8 classifiers), which correspond to combinations shown in Supplementary Figure 7 and Supplementary Figure 9. Row colors code for classification method as indicated in the legend. Methods EN-0.10, EN-0.50 and EN-0.90 were grouped under EN.

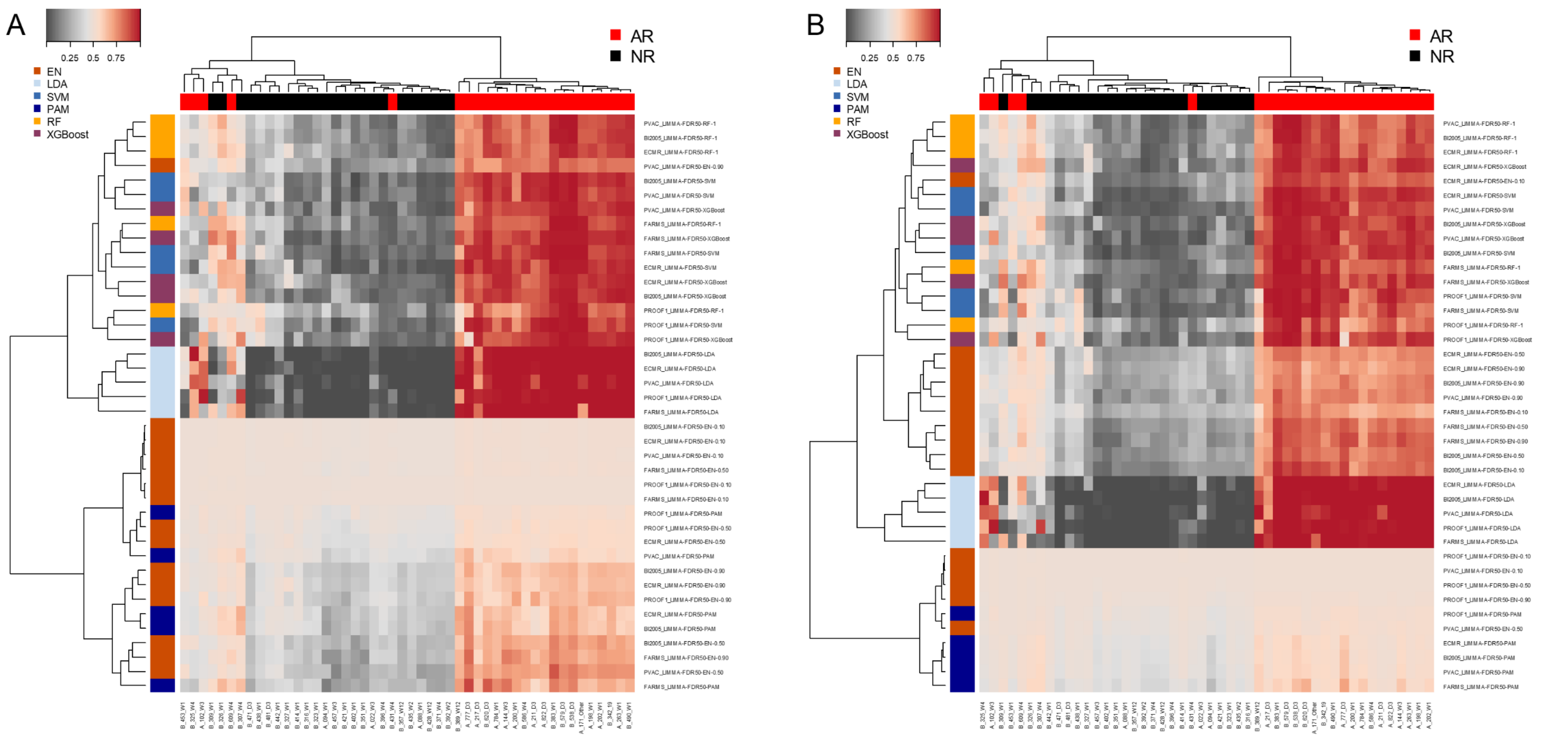

Supplementary Figure 11

Supplementary Figure 11. Most common classifier panel probe-sets. Frequency of top probe-sets/genes observed at least 25% over the complete set of 200 classifier panels identified in the analysis. Probe-set to gene mapping based on NetAffy annotations (v36; 2016).

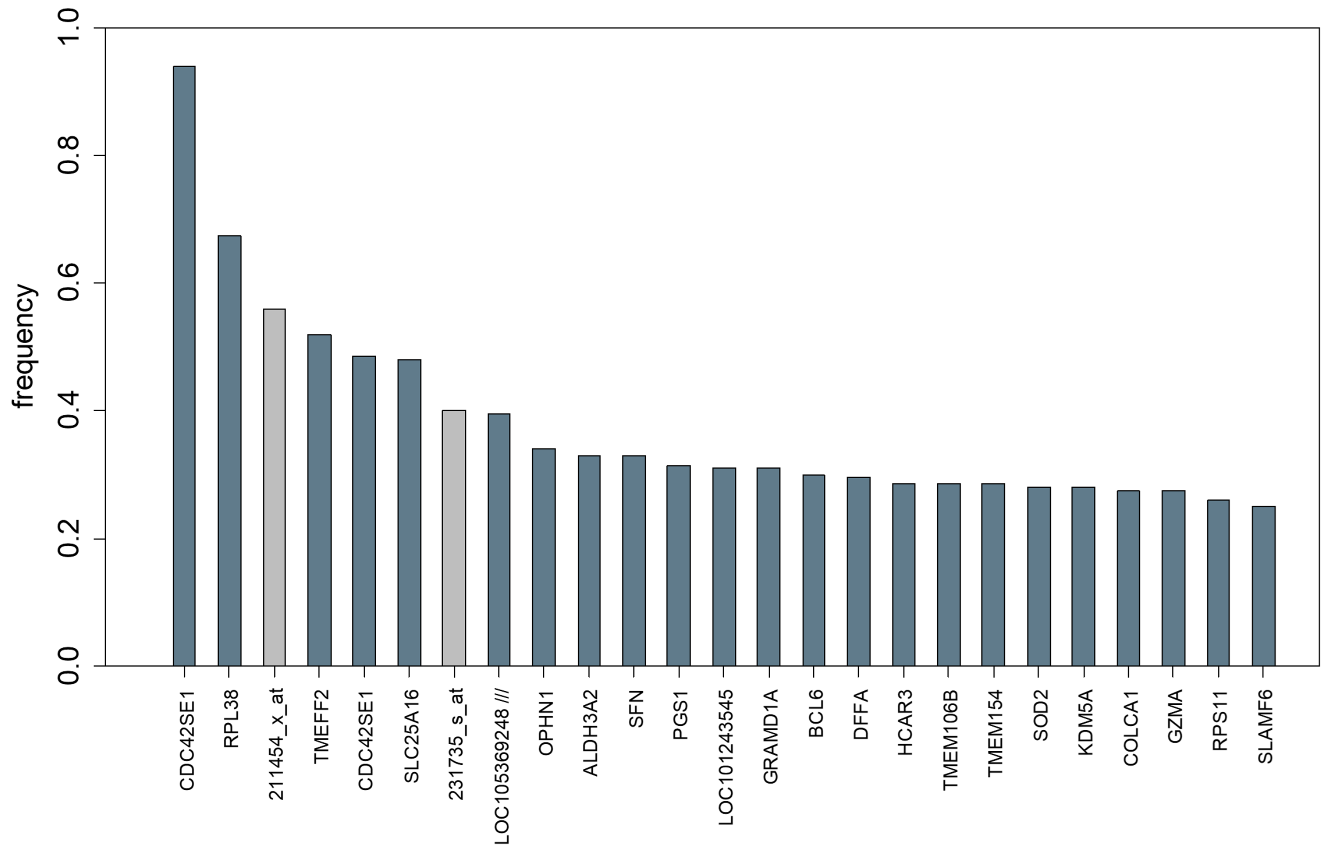

Supplementary Figure 12

Supplementary Figure 12. Most common classifier panel probe-sets by pre-filter. Shown are frequencies for finding specific probe-sets over 40 classifier panels (five ranking/filtering methods & eight classification methods). Each plot shows frequencies for a particular pre-filter approach as indicated. All analyses were based on the AUC one-standard error selection criteria. The number of probe-sets observed in at least 25% of 40 panels and the overall number of unique probe-sets were: ECMR (55; 640), PAM (55; 196), BI2005 (50; 273), FARMS (51; 164) and PROOF1 (43; 75).

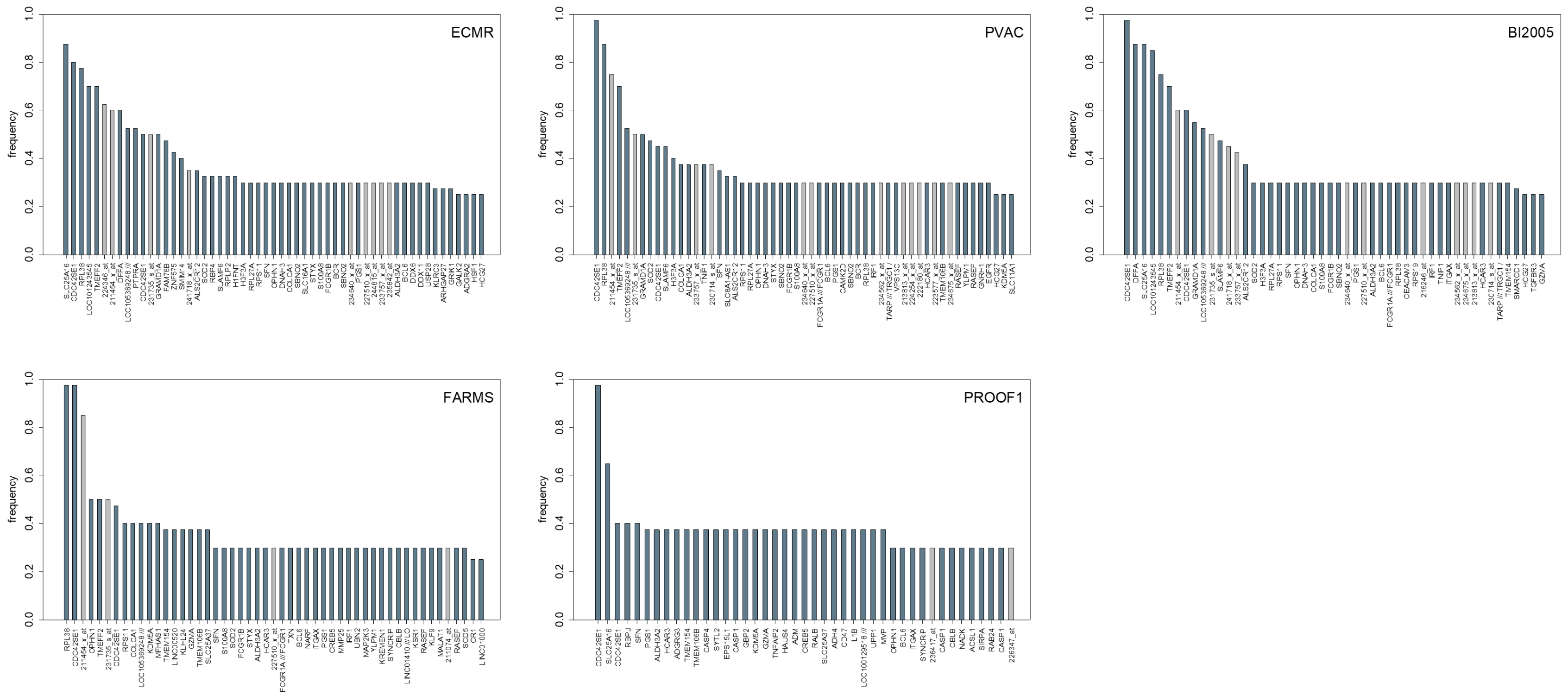

Supplementary Figure 13

Supplementary Figure 13. Most common classifier panel probe-sets by classifier. Shown are frequencies for finding specific probe-sets over 25 classifier panels (five pre-filter methods times five ranking/filtering methods). Each plot panel shows frequencies for a particular classification method as indicated. All analyses were based on analysis on the AUC one-standard error selection criteria. Results for SVM, RF and XGBoost are the same because they do not apply features selection and are shown in the top left panel for a cutoff fraction of 50% which returned 40 of 688 unique probe-sets over 25 panels. For the other methods, the number of probe-sets that are observed in at least 25% of 25 panels and the overall number of unique probe-sets were: LDA (8; 45), PAM (4; 33), EN-0.10 (8; 30), EN-0.50 (8; 45) and EN-0.90 (8; 35).

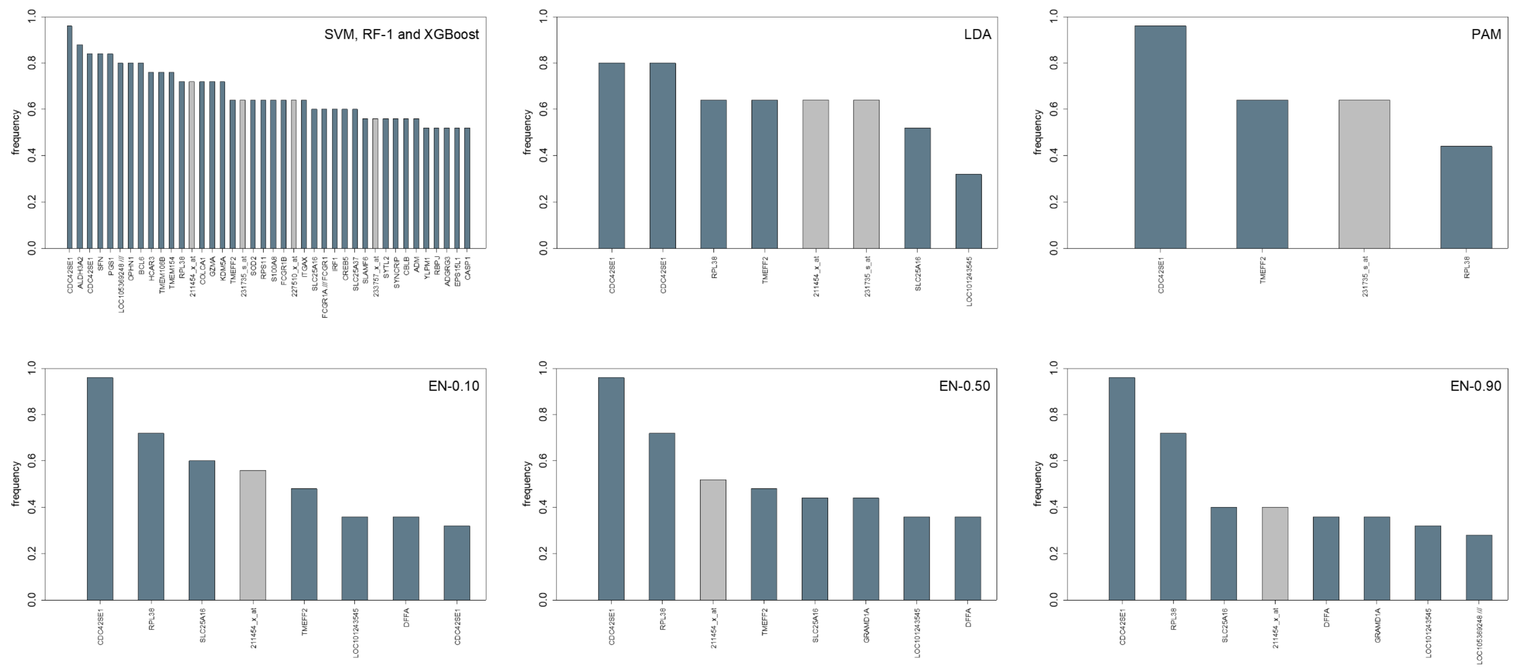

Supplementary Figure 14

Supplementary Figure 14. Most common classifier panel probe-sets by rank/filter. Shown are frequencies for finding specific probe-sets over 40 classifier panels (five pre-filter methods times eight classification methods). Each plot panel shows frequencies for a particular uni/multivariate ranking and filtering approach as indicated. All analyses were based on analysis on the AUC one-standard error selection criteria. The number of probe-sets observed in at least 25% of the 40 panels and the overall number of unique probe-sets were: FDR50 (24; 134), FDR0.10.RFE50 (21; 178) and FC0.5.TOP50 (27; 122). For the COMBO0.05 and FDR0.10.FC0.5 methods, a higher fraction threshold of 35% was used so fewer probe-sets would be selected to fit the display, resulting in: COMBO0.05 (29; 468) and FDR0.10.FC0.5 (57; 398).

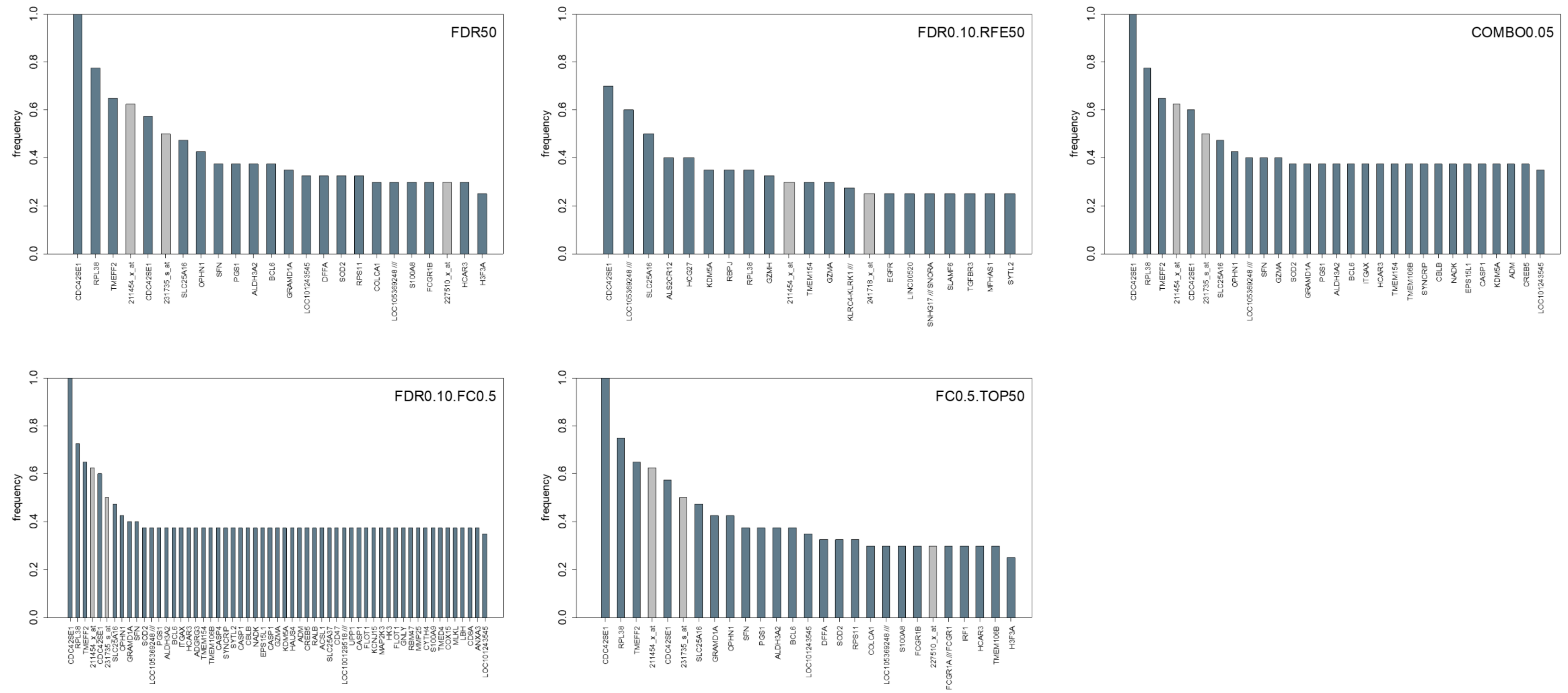

Supplementary Figure 15

Supplementary Figure 15. Score distributions for EN-0.10, LDA, PAM and XGBoost. Boxplots highlight the differences in score distributions for classification method EN-0.10 (top left), LDA (top right), PAM (bottom left) and XGBoost (bottom right). Boxplots for each sample summarize 25 probabilities from 5 pre-filter methods times 5 ranking/filtering approaches. Each of the 25 probabilities is an average of 5 partitions used in CV.

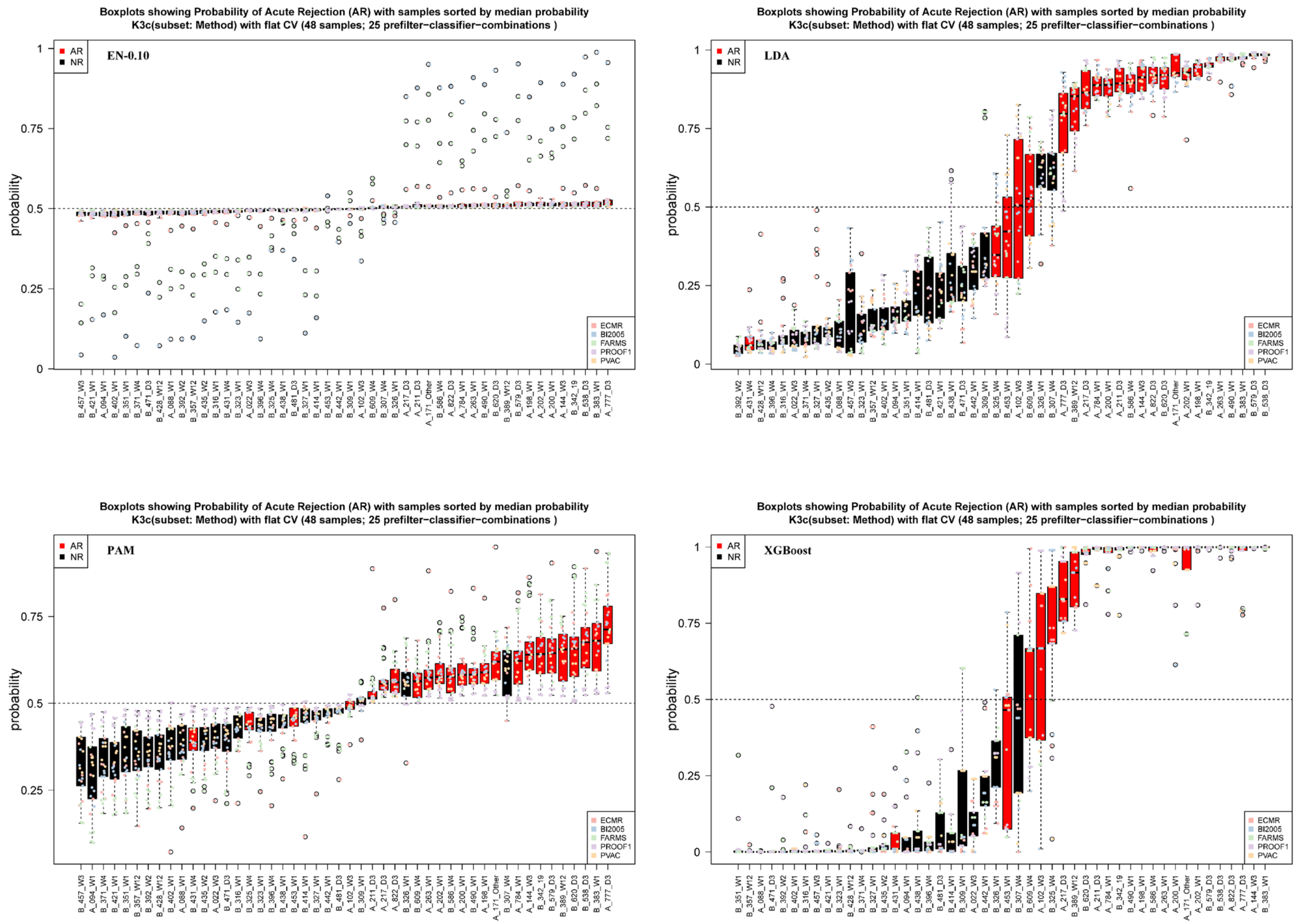

Supplementary Figure 16

Supplementary Figure 16. Performance distribution, scatter and correlation (AUC_one_se_max). Shown are density distribution (diagonal), scatter plots (lower diagonal) and correlations (upper diagonal) for four performance measures derived in nested CV for 5 pre-filters, 5 uni/multi-variate filters and 8 classifiers (using one-SE of maximum AUC for tuning). Colors represent either pre-filter method (for the 4x4 panels on the left) or classifier method (for the 4x4 panels on the right).

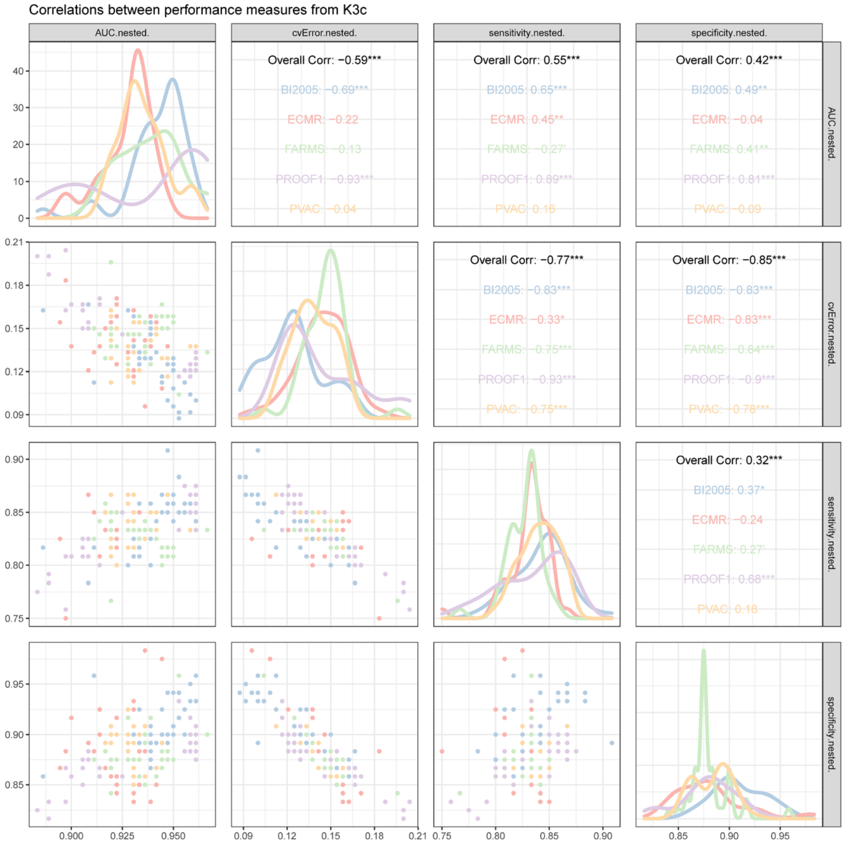

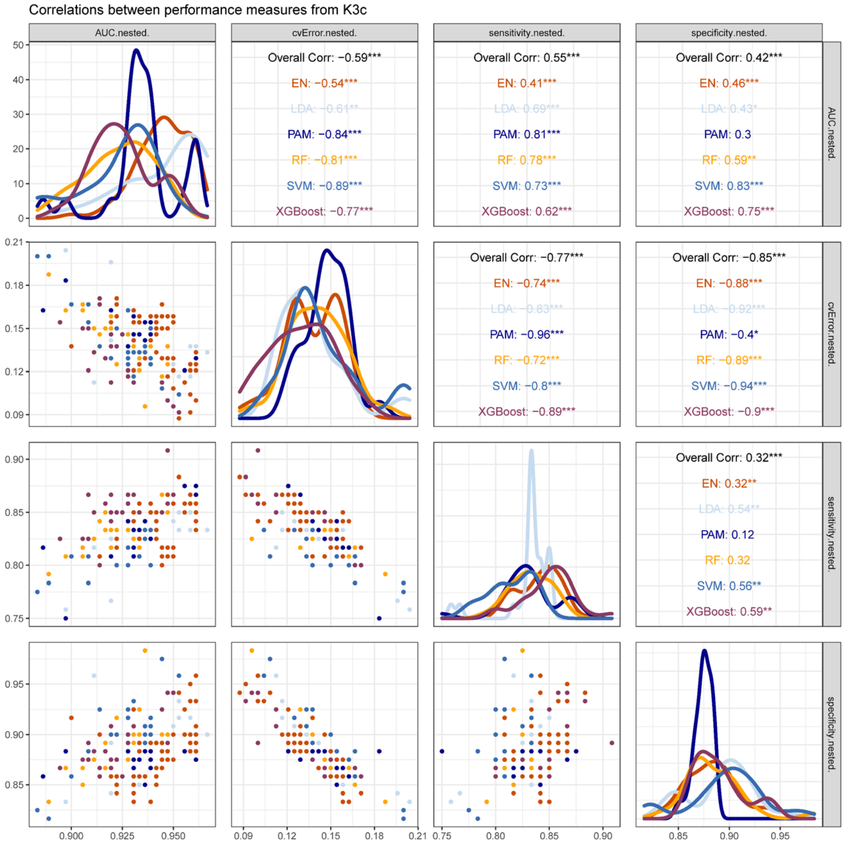

Supplementary Figure 17

Supplementary Figure 17. Feature and performance distribution, scatter and correlation (AUC_one_se_max). Shown are density distribution (diagonal), scatter plots (lower diagonal) and correlations (upper diagonal) for the number of features and performance measures derived in nested CV for 5 pre-filters, 5 uni/multi-variate filters and 5 feature-selecting classifiers (using one-SE of maximum AUC for tuning). Colors represent either pre-filter method (for the 4x4 panels on the left) or classifier method (for the 4x4 panels on the right).

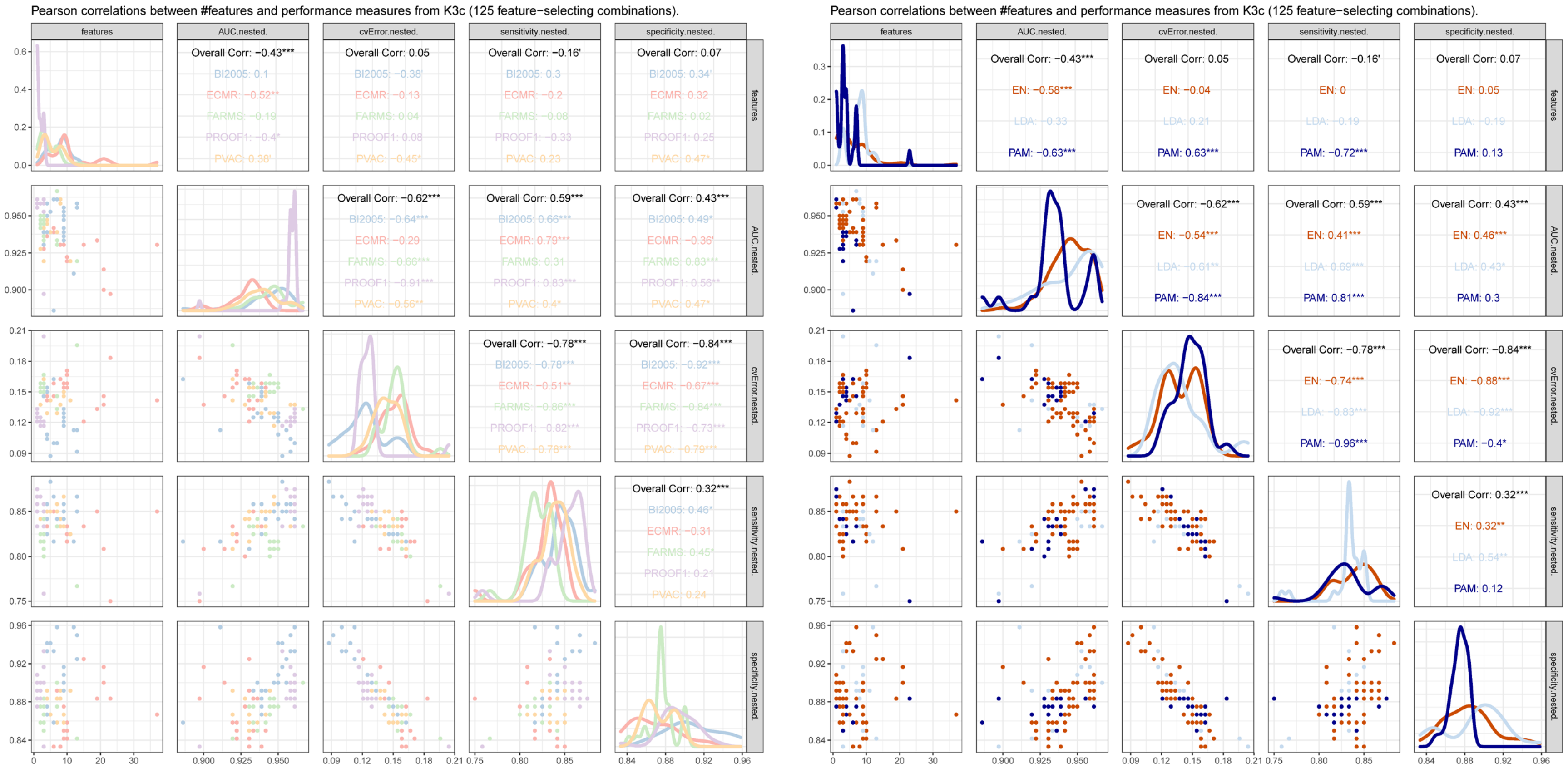

Supplementary Figure 18

Supplementary Figure 18. Reactome pathway enrichment analysis. Enrichment map of the Reactome pathway enrichment analysis using genes with FDR<0.1 of the five different pre-filters as input. The graph visualizes which pathways have been identified using multiple pre-filters and how those pathways interact.

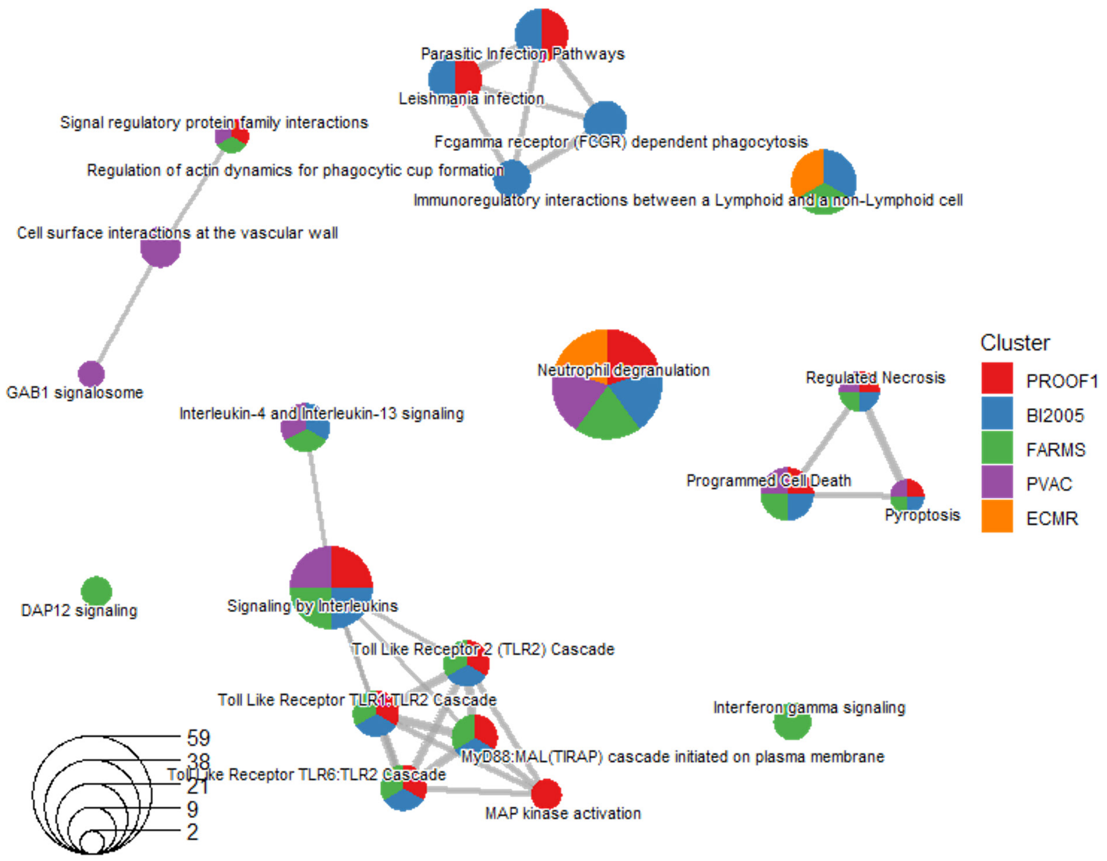

Supplementary Figure 19

Supplementary Figure 19. ROC-curves (multi-partition, flat CV). Comparison of performance (ROC-curves) by prefilter for each of 40 method combinations and outer 8-fold CV over 5 partitions. Colors indicate prefilter method.

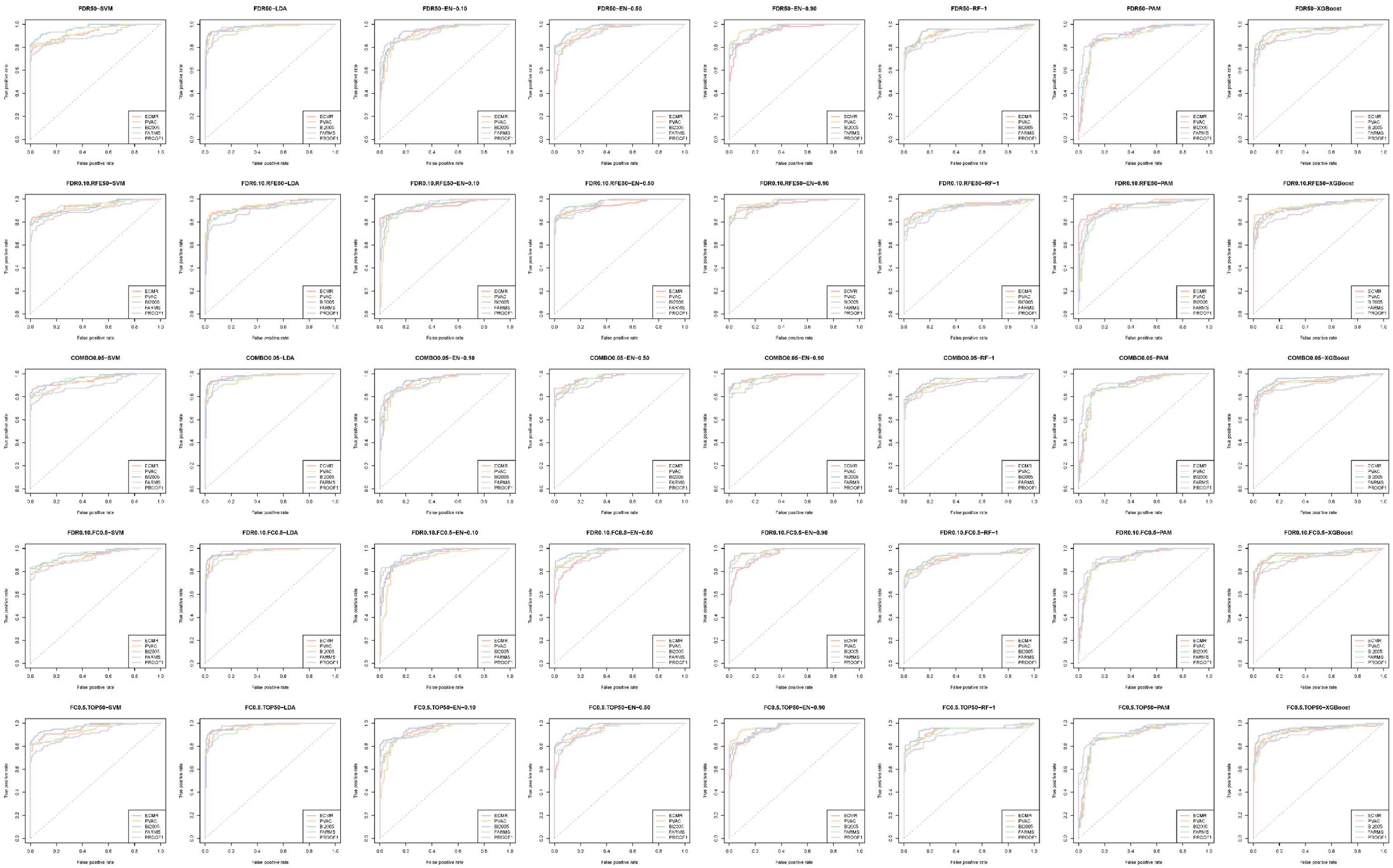

Supplementary Figure 20

Supplementary Figure 20. ROC-curves (multi-partition, nested CV). Comparison of performance (ROC-curves) by prefilter for each of 40 method combinations and nested 8-fold CV over 5 partitions. Colors indicate prefilter method.

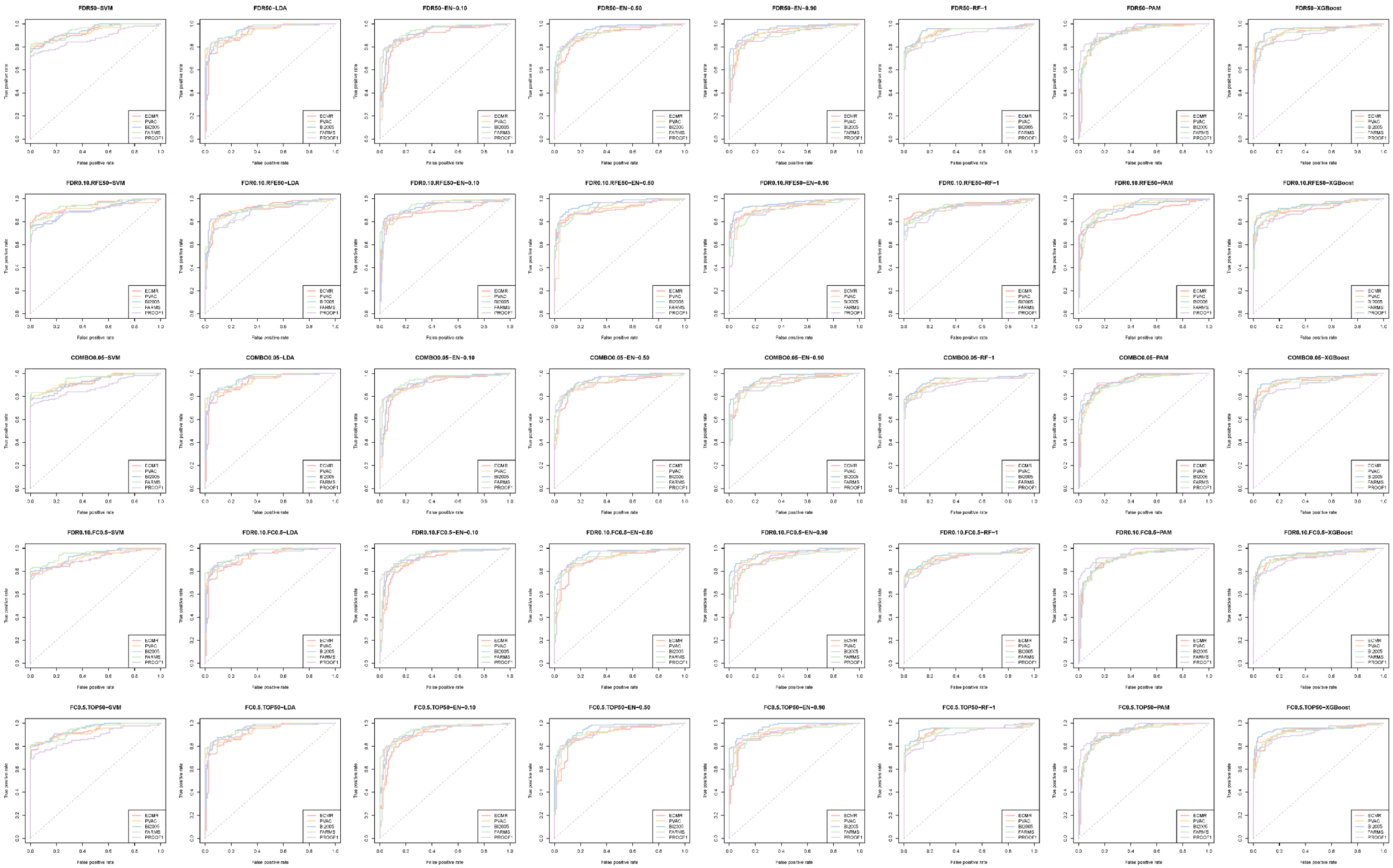

Supplementary Figure 21

Supplementary Figure 21. Overview of outer loop CV. Schematic overview of outer loop cross-validation for five partitions of the 48 samples used in the analysis.

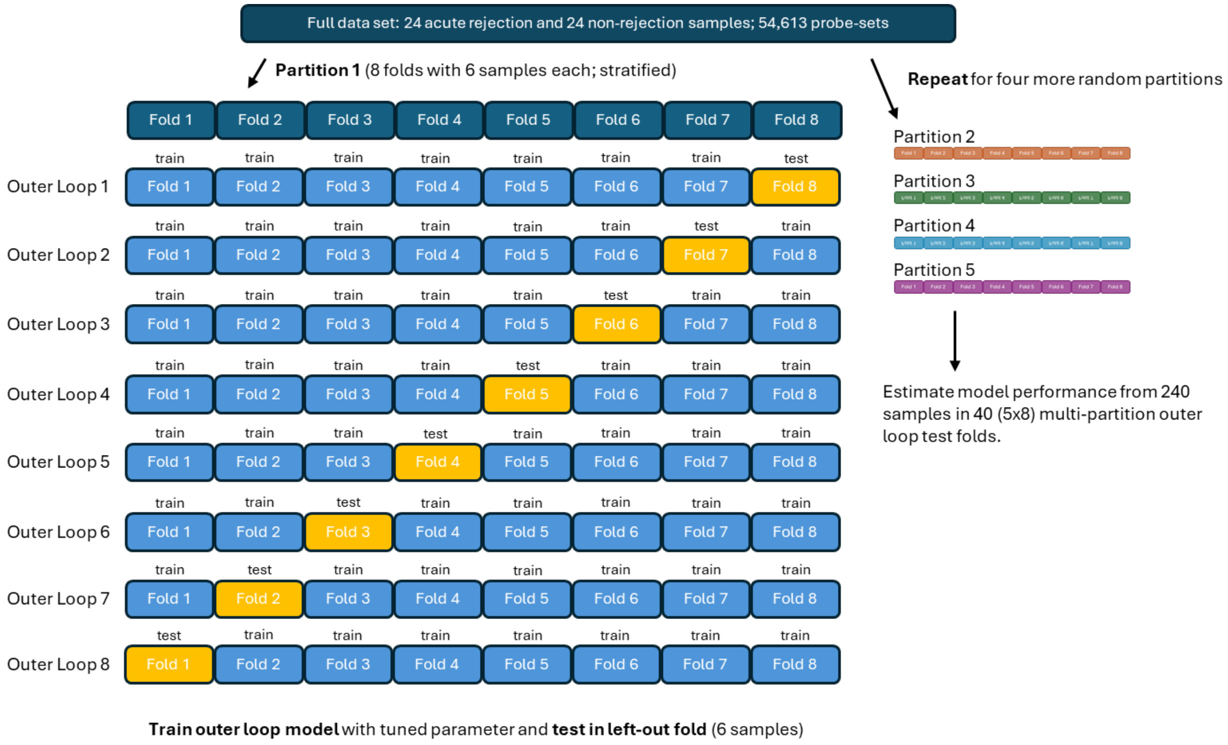

Supplementary Figure 22

Supplementary Figure 22. Overview of inner loop CV. Schematic overview of inner loop cross-validation setup for one outer loop over five partitions.

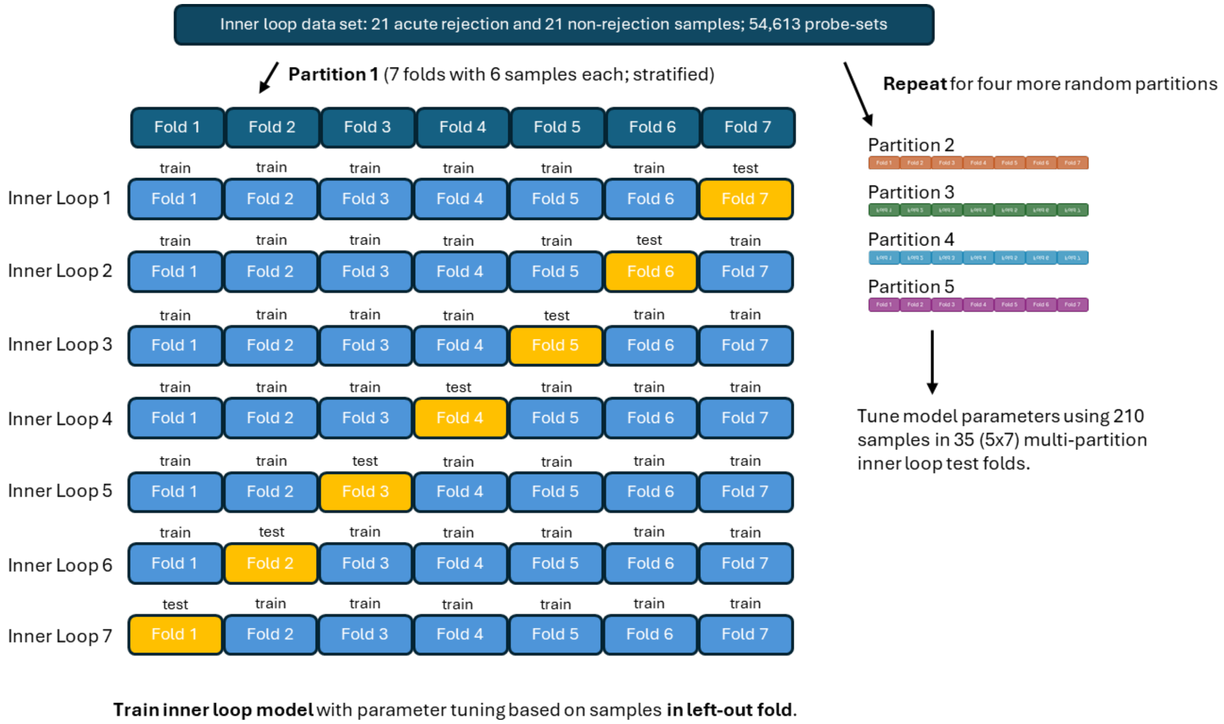

Supplement: Supplementary file 1 [file Supplementaryfile1.pdf]
